# Rational design of carbon nitride photocatalysts by identification of cyanamide defects as catalytically relevant sites

**DOI:** 10.1038/ncomms12165

**Published:** 2016-07-08

**Authors:** Vincent Wing-hei Lau, Igor Moudrakovski, Tiago Botari, Simon Weinberger, Maria B. Mesch, Viola Duppel, Jürgen Senker, Volker Blum, Bettina V. Lotsch

**Affiliations:** 1 Max Planck Institute for Solid State Research, Heisenbergstraße 1, 70569 Stuttgart, Germany; 2 Department of Chemistry, University of Munich, Butenandtstraße 5-13, 81377 Munich, Germany; 3 Department of Mechanical Engineering and Materials Science, Duke University, Durham, North Carolina 27708, USA; 4 Inorganic Chemistry III, University of Bayreuth, Universitätsstraße 30, 95447 Bayreuth, Germany; 5 Nanosystems Initiative and Center for Nanoscience (CeNS), Schellingstraße 4, 80799 Munich, Germany

## Abstract

The heptazine-based polymer melon (also known as graphitic carbon nitride, g-C_3_N_4_) is a promising photocatalyst for hydrogen evolution. Nonetheless, attempts to improve its inherently low activity are rarely based on rational approaches because of a lack of fundamental understanding of its mechanistic operation. Here we employ molecular heptazine-based model catalysts to identify the cyanamide moiety as a photocatalytically relevant ‘defect'. We exploit this knowledge for the rational design of a carbon nitride polymer populated with cyanamide groups, yielding a material with 12 and 16 times the hydrogen evolution rate and apparent quantum efficiency (400 nm), respectively, compared with the unmodified melon. Computational modelling and material characterization suggest that this moiety improves coordination (and, in turn, charge transfer kinetics) to the platinum co-catalyst and enhances the separation of the photogenerated charge carriers. The demonstrated knowledge transfer for rational catalyst design presented here provides the conceptual framework for engineering high-performance heptazine-based photocatalysts.

## Abstract (web summary)

Graphitic carbon nitride is a promising hydrogen evolution photocatalyst, although there is limited understanding of its mechanistic operation. Here, the authors employ molecular heptazine-based model catalysts to identify catalytically relevant defects and to rationally design a highly active carbon nitride photocatalyst.

## Graphical abstract

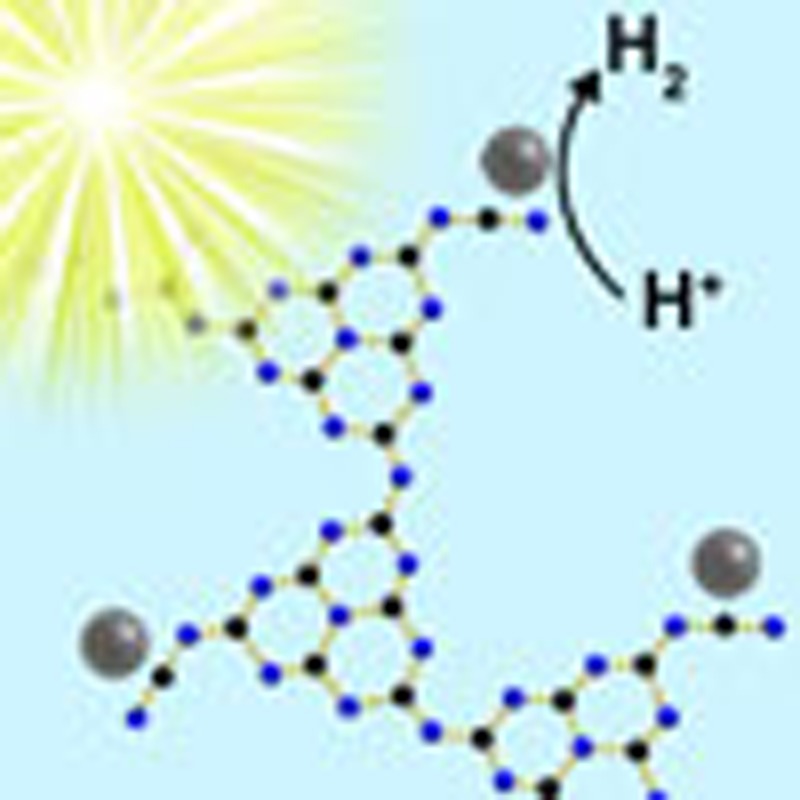

Photocatalytic hydrogen evolution is considered to be a promising technology for the direct capture and storage of solar energy, since the energy stored in the hydrogen–hydrogen bond can be extracted with high efficiency without producing environmentally harmful by-products^1^. Numerous materials^2^ have been reported since the seminal report of Honda and Fujishima^3^ on water splitting photocatalysed by titanium dioxide; yet none of them are at a stage for industrial deployment. The common disadvantages with most of these photocatalytic materials are as follows: (1) low activity under visible light; (2) costliness associated with rare elements and/or synthetic processes; and (3) lack of chemical stability.

One promising photocatalytic material is melon, also referred to erroneously in the literature as graphitic carbon nitride (g-C_3_N_4_)^4^. It is easily prepared from inexpensive precursors (urea, dicyandiamide or melamine) and has a suitable band gap and potential for water reduction/oxidation under visible light, good chemical stability and, finally, molecular tunability. Its low activity is addressed primarily through texturization for increased surface area^5^,^6^,^7^, copolymerizing with organic^8^,^9^,^10^ or inorganic^11^,^12^,^13^ dopants for tuning its optoelectronic properties, compositing with (semi)conductors for enhanced charge separation^14^, and employing hetero- or homogeneous co-catalysts to improve interfacial charge transfer^15^,^16^. Our approach is to gain an intimate understanding of the structure–activity relationship of these carbon nitride materials, and then transferring this knowledge to the rational design of improved, that is, inherently more active, photocatalysts. Following the accepted postulate that ‘…surface terminations and defects seem to be the real active sites…'^17^, we have previously demonstrated that oligomers outperform polymers in terms of photocatalytic hydrogen evolution rate^18^. Here we aim to elucidate the identities of the catalytically relevant surface terminations of carbon nitrides by employing heptazine-based molecules as model catalysts. This methodology, employed to circumvent complication from surface heterogeneity and structural ambiguity in heterogeneous catalysis research^19^,^20^,^21^,^22^,^23^, is particularly suitable for studying melon which, because of its amorphous nature and lack of solubility, could only be structurally resolved nearly two centuries after its discovery. Its photophysical properties resemble those of a quasimonomer, suggesting exciton localization within each heptazine unit of the polymer^24^,^25^, thereby justifying the use of heptazine molecules as model candidates. In previous examples demonstrating this approach, variations of spectroscopic, electrochemical and photocatalytic properties of melon have been investigated as a function of polymer length using di- and trimeric heptazine-based oligomers^18^,^26^. In this work, we first screen for photocatalytic activity for hydrogen evolution in the heptazine molecules and complexes shown in Fig. 1, which differ in the functional group at the 2, 5 and 8 positions of the heptazine core. Having identified catalytically relevant moieties, we further demonstrate the process of rational catalyst design by introducing one of these functionalities—the cyanamide group—into the polymer chain to enhance its intrinsic photocatalytic activity.

## Results

### Model photocatalysts

Functional groups that are identified as potentially relevant for catalysis include not only amines (primary, secondary or tertiary) but also the cyanamide moiety and oxygen-bearing groups. Cyanamide may occur because of incomplete heptazine cyclization based on some proposed mechanisms for heptazine formation or thermal depolymerization (Supplementary Fig. 1)^27^,^28^,^29^, while oxygen-bearing groups may be present from impurities in the precursors (for example, ureidomelamine is a significant impurity in melamine^30^) or oxygen contamination through trace water during high-temperature syntheses in air. Detailed discussions on the selection of the model catalysts, their characterization (including spectral properties) and the screening protocols are provided in the Supplementary Discussion and Supplementary Figs 2–8. We emphasize that these groups have largely been overlooked to date as catalytically relevant in the literature, and most publications generally depict g-C_3_N_4_ as an idealized two-dimensional polymer of heptazine units bridged (2° and 3°) and terminated solely by amines (1°). Note that the photocatalytic models were screened only for the presence of activity but were not compared for their hydrogen evolution rates quantitatively since the models differ in their spectral profile and solubility/dispersibility in aqueous solution.

Using the standard protocol as outlined in the Supplementary Information (see Supplementary Fig. 9 for the spectra of the irradiation source), photocatalytic activity was observed for the secondary amine (**2**), the cyanamide–platinum complex (**5k**, with and without added H_2_PtCl_6_) and the oxygen-containing models (**6** and **7**); see Supplementary Figs 10–11 for the hydrogen evolution plots. These results thus justify our hypothesis that functionalities from incomplete heptazine cyclization (cyanamide) and impurities (oxygen-bearing functional groups) can also be catalytically relevant and may, in fact, be some of the catalytically active ‘defects' cited in the literature^17^. Activity was also observed for the heptazine tribenzoic acid (**9**) but not for the tolyl equivalent (**8**), suggesting the necessity of a coordinating group. While the above catalytically active species represent the first molecular model compounds for carbon nitride photocatalysts, Pt-melonate is distinct in its well-defined coordination mode and in the fact that it produces hydrogen both with and without addition of H_2_PtCl_6_ (in-depth characterization and discussion of the results shown in Supplementary Figs 12–14 and Supplementary Discussion). Pt-melonate therefore provides a unique platform for investigating charge transfer from the heptazine core to the solution, via the platinum centre, for hydrogen evolution.

From the screening of these models, we attempted to identify some predictive descriptors based on properties intrinsic to the molecule itself, namely frontier orbitals and their energy levels, which may lead to the presence of activity, using density functional theory (DFT) adapted^31^,^32^ to approximate higher-level *G*_0_*W*_0_ quasiparticle calculations for the energy levels of the isolated models (Supplementary Fig. 15, see Supplementary Methods for details regarding the calculations using ‘FHI-aims' all-electron code^33^,^34^). In our calculations, the photocatalytic activity was not found to correlate with the highest occupied and lowest unoccupied molecular orbitals (HOMO/LUMO) locations, the energy levels or the energy gaps. The absence of a correlation is most evident for the melem-derived series of models **1**, **2** and **3**, which differ by the successive attachment of ethyl groups to the amine side group but are electronically very similar, suggesting that other intrinsic or extrinsic factors such as intermolecular interactions and steric hindrances are at play. Combining all the screening results, we infer that a required (although not necessarily sufficient) criterion for catalytic activity may be the presence of a coordinating group, which provides the ligating linkage to the platinum centres to facilitate the transfer of the photoexcited charge from the heptazine core. This criterion, however, can be complicated by intra-/intermolecular bonding in the condensed phase, where hydrogen bonding or steric hindrance locks up the availability of the ligating moieties. The models where this may be the case are crystalline melem and melon, where all amine groups are blocked by hydrogen bonding, as well as tri(diethylamino)heptazine, where the ethyl groups hinder coordination between the 3° amine and the platinum.

### Cyanamide functionalization of melon

Armed with these insights, we next demonstrate the rational design of a highly active carbon nitride photocatalyst by deliberately populating the polymer with one such catalytically relevant group identified in the screening experiment, namely the cyanamide moiety. The rationale for choosing this particular functional group comes from matrix-assisted laser desorption/ionization–time of flight (MALDI–TOF) measurements, which suggest that cyanamide groups are indeed present already in native amorphous melem synthesized from melamine under argon at ambient pressure (Supplementary Fig. 3 and Supplementary Table 1). The NCN moiety may therefore be innate in small amounts to carbon nitrides when synthesized under certain conditions and may result from polymerization/depolymerization equilibria involving cyanamide as suggested for the formation of melem (Supplementary Fig. 1).

In our prototype design, functionalization of melon with cyanamide was achieved following the KSCN salt melt synthesis for potassium melonate, except that the yellow insoluble solid was isolated, following the literature mechanism in Supplementary Fig. 16. This residue (notated henceforth as ‘KSCN-treated melon') exhibited a steady hydrogen evolution rate of 24.7 μmol h^−1^ after more than 70 h (31.2 μmol h^−1^ in the first 4 h) of AM 1.5 irradiation at an optimum platinum loading of 8 wt% using H_2_PtCl_6_ as the platinum source and 10 vol% of aqueous methanol as electron donor (Fig. 2; platinum optimization in Supplementary Fig. 17). We observed a colour change of the catalyst suspension from yellow to turquoise or blue during irradiation, suggestive of a long-lived excited state. The nature of this state as well as the implications for photocatalysis will be expounded upon in forthcoming studies. Apparent quantum efficiencies (AQEs) were estimated to be 9.3% under 400 nm irradiation and 0.34% across the entire air mass (AM) 1.5 spectrum. In accordance with our hypothesis, this vastly outperformed amorphous melon, which has a hydrogen evolution rate of 2 μmol h^−1^ at 1 wt% optimized platinum loading under identical conditions and an AQE of 0.5% at 400 nm and 0.027% for AM 1.5. Negligible activities under 500 nm irradiation for both samples are consistent with their absorption profile (Fig. 2). While an internal quantum efficiency of 26.5% has been reported for heptazine^35^ photocatalysts recently, these experiments employed triethanolamine, which as an electron donor increases the hydrogen evolution rate by 3.5 times compared with methanol (Fig. 2d) on account of its more negative redox potential^36^. We, nonetheless, did not select triethanolamine (nor the even more active sodium oxalate) to optimize our AQE, since it suffers from some disadvantages (for example, light sensitivity, optical impurity, *E*°=−600 mV) not present for methanol (*E*°=+200 mV). In stating these, we consider our AQE of 9% competitive with the above literature values, since they do not exceed our value by more than three times. In this sense, the activities observed for KSCN-treated melon are among the best reported for carbon nitride photocatalysts, although in our case neither compositing nor texturization was applied. A comparison with other visible light photocatalysts, based on their AQE as suggested^37^, is provided in Supplementary Table 2 and demonstrates that KSCN-treated melon is competitive with several state-of-the-art materials. Moreover, its performance is a significant step towards the record holders in inorganic visible light photocatalysts, such as CdS and AgInZn_7_S_9_; note that these materials also suffer from drawbacks not present in the carbon nitride photocatalysts (for example, instability and toxicity).

Negligible changes of the optoelectronic properties of KSCN-treated melon compared with pristine melon based on their spectral profiles and Fermi levels (valence band region of X-ray photoelectron spectroscopy (XPS) in Supplementary Fig. 18) rule out that changes in band energies are the principal factors for KSCN-treated melon's high performance.

To explore the structural and compositional features of KSCN-treated melon leading to its high photocatalytic performance, we characterized the material by solid-state magic angle spinning NMR, FTIR (Fourier transform infrared) and Raman spectroscopies, X-ray diffraction, SEM (scanning electron microscopy) and TEM (transmission electron microscopy; Fig. 3 and Supplementary Figs 19 and 20, details of NMR results summarized in Supplementary Table 3) as well as elemental analysis (Supplementary Table 4), XPS (Fig. 4), physisorption (Supplementary Fig. 21, results summarized in Supplementary Table 5) and thermogravimetric analysis coupled with mass spectroscopy (TGA-MS; Supplementary Fig. 22). Note that NMR spectroscopy was performed on the material prepared from both KSCN and enriched KS^13^C^15^N (for FTIR comparing the two, see Supplementary Fig. 19). The heptazine core is confirmed by its characteristic infrared vibration at 804 cm^−1^, the Raman ring vibrations in the range 1,653–1,160 cm^−1^ (ref. 38), the ^13^C NMR shifts at 157 (C2) and 163 ppm (C3) and the ^15^N shifts for the central (N3) and peripheral nitrogens (N4) at −225 and −160 to −205 ppm, respectively, as well as the *sp*^2^ carbon (288.4 eV) and nitrogen (398.8 eV) signals in the XPS. Its polymeric nature is shown by the presence of the bridging 2° amine as observed in the C–N IR bending mode (1,311 and 1,221 cm^−1^), the ^15^NH (N2) NMR signal at −243 ppm and the XPS signal at 401.1 eV. The central N3 ^15^N NMR signal of the heptazine ring at −225 ppm, identical to the polymer^4^ and dissimilar to the monomer^39^ (−235 ppm) and oligomers, (−225<*δ*<−235 ppm)^18^ as well as the absence of blue shift in the UV–vis spectrum are evidence for the polymeric nature of KSCN-treated melon^18^. The cyanamide group is evidenced by the IR and Raman band at 2,177 cm^−1^ assigned to vibrations involving C≡N stretch^40^, and the corresponding ^13^C signal is observed at 118.2 ppm (C1) and ^15^N signals at −175 (N1) and −276 ppm (N5), consistent with literature values for melonate (potassium^38^ and other^41^ metal salts), organic cyanamide compounds^42^ and tricyanomelaminates^43^,^44^. The reasons for the lower than expected intensities for the cyanamide NMR signals are discussed in greater depth in the Supplementary Discussion; however, the postulated structure is nonetheless supported by elemental analyses, with the C:N atomic ratio found to be 0.70 and roughly 40% potassium exchanged with protons. Sulfur is detected with inductively coupled plasma atomic emission spectroscopy (ICP-AES) in trace amounts (0.17 wt%) but remained undetected with the surface-sensitive technique XPS (Fig. 4), and seemed not to adversely affect the photocatalytic activity. We therefore conclude that it is either present in negligible quantity or is hidden in the bulk of the catalyst particles and, hence, is unlikely to participate in surface reactions^45^. The trace amount of sulfur acting as a ‘dopant' is also considered unlikely, as this would typically entail changes in optoelectronic properties in excess of 0.1 eV, which is not the case here^11^.

Compared with the X-ray diffraction of amorphous melon with the stacking and in-plane periodicities^4^,^46^ at 27.56° 2*θ* (3.23 Å) and 12.8° 2*θ* (6.9 Å), respectively, KSCN-treated melon has a denser layering (28.26° 2*θ*, 3.16 Å) and exhibits in-plane periodicities at 11.0 Å (8.03° 2*θ*) and 9.02 Å (9.82° 2*θ*). The 11 Å lattice fringes are also observed in the bright-field images and its fast Fourier transform (FFT), as well as in the selected area electron diffraction analysis. Sorption measurements indicate that most of the pore volume is in the micro- and low-mesoporosity range (Supplementary Fig. 21 for pore size distribution histogram) with the larger mesopores attributed to interparticle spacing. Although the Brunauer–Emmett–Teller (BET) surface area and pore volume are, respectively, 3.4 and 2.7 times higher than those of amorphous melon (Supplementary Table 5), the intrinsic activity of KSCN-treated melon based on normalization of activity to BET surface area still outperforms amorphous melon's activity—22.2 μmol H_2 _h^−1 ^m^−2^ versus 7.5 μmol H_2 _h^−1 ^m^−2^—which we attribute to the rational insertion of the cyanamide group.

### Catalytic role and evolution of the cyanamide moiety

Characterizations of the spent catalyst after 100+ h of irradiation (Fig. 4, Supplementary Figs 23 and 24 and Supplementary Table 6, interpreted in greater details in the Supplementary Discussion), indicate partial hydrolysis of the cyanamide moiety to urea and may account for the drop of the hydrogen evolution rate of 31.2 μmol h^−1^ in the first 4 h to the stable rate of 24.7 μmol h^−1^ thereafter. The FTIR, NMR and XPS spectra of the spent catalyst are nearly identical to those of melon terminated by urea, which can be prepared by acid hydrolysis of the KSCN-treated melon. We observe that the cyanamide signal in the FTIR (2,181 cm^−1^) decreases significantly in the initial 4 h of the photocatalytic reaction (Supplementary Fig. 24), and then drops off slowly as the reaction proceeds. Our rough estimate, based on changes in the ratio of FTIR intensities of the cyanamide (2,181 cm^−1^) to the heptazine signal (805 cm^−1^), is that 67% of the cyanamide has been hydrolysed in the spent catalyst after 100+ h reaction. We nonetheless emphasize that, for this prototype catalyst designed from our model screening, the final steady rate for hydrogen evolution is still 80% that of the initial rate and is significantly higher than that of amorphous melon, as outlined above. In other words, despite significant loss of the cyanamide, its hydrolysis product still exhibits high photocatalytic activity for hydrogen evolution. We rationalize this observation by the continued strong interaction between Pt and the carbon nitride at the Pt-CN_*x*_O_*y*_ heterointerface, before and even after hydrolysis. As consistent with previous investigations of melonate complexes, the cyanamide moiety is a better ligand compared to materials in which the primary amines and the heptazine nitrogens are present (for example, as in melem)^47^. Experimentally, this interaction is evidenced by the presence of both Pt^0^ and Pt^II^ in the XPS of the spent KSCN-treated melon, as well as that of melon, which is consistent with literature observations^48^. However, the lower than expected binding energies (B.E.) for Pt^0^ at 71.1–71.2 eV and for Pt^II^ at 72.4 eV are more akin to those of platinum on strongly interacting supports such as TiO_2_ or CeO_2_ (70.4–70.8 eV for Pt^0^ and 71.5–72.5 eV for Pt^II^)^49^,^50^,^51^,^52^,^53^,^54^ rather than on less interacting supports such as SiO_2_ or carbon (71.6–71.9 eV for Pt^0^ and >72.7 eV for Pt^II^)^51^,^52^,^55^,^56^,^57^. Together with the minute shifts to higher B.E. for the N_1*s*_ and C_1*s*_ signals in the spent melon, these XPS results allude to a metal–support interaction (MSI) in these carbon nitride photocatalysts with polarization of electron density from the support to the platinum, based on the direction of these shifts. Shifts of all signals larger than spectrometer resolution, except for the adventitious carbon signal used for calibration, lend weight that the observed changes are not calibration errors. This MSI effect has also been attributed to improved platinum electrocatalytic activities^52^,^54^,^58^,^59^ for reactions such as the oxygen reduction reaction. Note that MSI has also been cited to be beneficial for methanol electro-oxidation using a PtRu electrocatalyst supported on a melon/carbon composite, although in these cases the two metals had higher B.E. compared to the findings when only carbon was used, indicating a loss of electron density in the metal particles^60^,^61^. Although the carbon nitrides are not as interacting as TiO_2_ and CeO_2_ based on the magnitudes of the shifts, this MSI effect provides a consistent rationale to the necessity of a coordinating group in the heptazine models or of ‘defects' in amorphous melon to exhibit photocatalytic activity. Importantly, the role of such ligating groups or defects may thus be to facilitate interactions between the polymer and the co-catalyst for efficient charge transfer.

### Computational modelling of the cyanamide functional group

In addition to its promotional effect, we explored computationally whether this anionic moiety increases local fluctuations of the electrostatic potential within the polymer, thus enhancing the charge separation. As an indicator of the possible charge separation tendency, we calculated the HOMO/LUMO locations for three different terminations of the same heptazine pentamer conformation using DFT as above (for computational details, see Supplementary Methods, Supplementary Figs 25–27 and Supplementary Table 7). Figure 5 shows the resulting orbitals for the pentamer terminated with NH_2_, proton-terminated NCN or potassium-coordinated NCN side groups, that is, three overall neutral molecules with increasing ionicity of the side group and counter charge. Obviously, the HOMO and LUMO for the case with K^+^ as the cation are well separated, significantly more localized and contained within different heptazine units of the pentamer, than those for the hydrogen-terminated case or the termination by NH_2_. We attribute the differences in preferential electron or hole locations on the pentamer to the stronger local electrostatic potential differences on different heptazine units because of the locations of the K^+^ cations. One consequence is the spatial separation of the electron–hole pair, thereby reducing their recombination and increasing their probability of interfacial charge transfer for the desired redox reaction. These results are similar to semiconductors with a built-in electric field, which lead to better charge separation and hence higher activity^62^,^63^. More significantly, the result implies that introducing an ionic moiety may be a valid strategy for enhancing charge separation in carbon nitrides. We also note again the observed colour change of the suspension during photocatalysis, which is attributed to a long-lived radical species. As exciton localization and charge separation have been considered to be the major limitation to the photocatalytic efficiency for carbon nitride-type materials^64^, our current work may open new avenues for rationally improving the photocatalytic activity of such systems.

## Discussion

Using heptazine-based molecular models for screening photocatalytic activity, we have identified catalytically relevant functional groups in amorphous melon. We then exploited this knowledge for rational catalyst design by populating amorphous melon with one of these groups, the anionic cyanamide moiety. This prototype catalyst evolves hydrogen at a stable rate at least 12 times higher than the unmodified melon and 18 times in terms of AQE at 400 nm. Characterization of the catalyst after photocatalysis and computational experiments suggest two roles of this moiety: (1) enhancing co-catalyst interactions and thus facilitating interfacial electron transfer to the platinum centres and (2) improving the separation of the photoexcited charges through built-in electrostatic potential differences across the heptazine polymer. In demonstrating this case of ‘defect engineering' with cyanamide, we emphasize that such moieties may be present in the prototype polymer melon formed under certain synthesis conditions, based on the TGA-MS and MALDI–TOF analyses of amorphous melem and melon. These findings provide the rationale for the wide variation of photocatalytic activity as the synthesis conditions and the precursors used are changed, namely the differences in the amount and type of catalytically relevant ‘defects' formed in the resulting material. Investigating the promotional role of such ‘defects' thus offers a yet unexplored avenue to improving the intrinsic photocatalytic activity of polymeric carbon nitride. The methodologies, mechanistic insights and process of rational catalyst design demonstrated herein can be adapted to studying other light-driven reactions (oxygen evolution or CO_2_ reduction), as well as exploring other possible functional groups non-native to melon, which ultimately would provide the blueprint for the design of highly active, customizable heptazine-based photocatalysts.

## Methods

### Synthesis procedures

Heating at temperatures above 300 °C was carried out in a custom-made resistive furnace, using a Eurotherm 2408 temperature controller. Where applicable, percentage molar yields are calculated assuming the ideal formula of C_6_N_9_H_3_ for melon. Crystalline melem (**1**)^39^, crystalline melon (**10**)^4^, amorphous melem^65^, amorphous melon^18^, tri(diethylamino)heptazine (**3**)^66^, potassium cyamelurate (**6**)^67^, cyameluric acid (**7**)^68^, tri(*p*-tolyl)heptazine (**8**)^69^, tri(*p*-benzoic acid)heptazine (**9**)^69^ and potassium melonate^38^ (**5**) were synthesized following literature methods and their characterizations are consistent with those previously reported.

Heptazine triphthalimide (**4**) was prepared following the literature method^65^, except that unreacted melem was not removed by Soxhlet extraction with nitromethane because of safety concerns.

Cyameluric trichloride was prepared following an adapted procedure^70^. Potassium cyamelurate (24.4 g) was refluxed in a mixture of PCl_5_ (6.81 g) and POCl_3_ (10 ml) until gas evolution has ceased. Unreacted PCl_5_ and POCl_3_ were boiled or sublimed off by heating under vacuum. The yellow product was not purified from the side product KCl as it is easier to remove in the subsequent syntheses. Yield 34.1 g (molar yield not provided as residual KCl was not removed). Characterization with FTIR is consistent with literature^70^.

Tri(ethylamino)heptazine (**2**) was synthesized by mixing under argon a solution of cyameluric trichloride (1.386 g) in tetrahydrofuran (THF, 20 ml, anhydrous) and ethylamine in THF (8.5 ml, 2 M L^−1^), and then refluxed for 2 h. The solvent and unreacted ethylamine were evaporated off and the resulting solid was re-dispersed in water, refluxed for 1 h, isolated by filtration, and then washed repeatedly with water and dried. The product was further purified by recrystallization from hot glacial acetic acid. Yield: 933 mg (62%). ^1^H NMR (DMSO): *δ*=2.50 (CH_2_), 1.07 ppm (CH_3_). FTIR: 3,222, 3,080, 3,029, 2,971, 2,933, 1,641, 1,571, 1,494, 1,433, 1,398, 1,373, 1,346, 1,308, 1,286, 1,178, 1,144, 1,100, 1,069 and 797 cm^−1^.

Transition and lanthanum metal complexes of melonate were prepared by mixing stoichiometric amounts of potassium melonate and the metal salt, both as aqueous solutions (20 mM). The metal salts employed are: (**5a**) AgNO_3_, (**5b**) CeCl_3_·6H_2_O, (**5c**) Co(NO_3_)_2_·6H_2_O, (**5d**) Cr(NO_3_)_3_·9H_2_O, (**5e**) Cu(AcO)_2_·H_2_O, (**5f**) Fe(NO_3_)_2_·nH_2_O, (**5g**) La(NO_3_)_3_·6H_2_O, (**5h**) Mn(AcO)_2_·4H_2_O, (**5i**) Nd_2_(SO_4_)_3_, (**5j**) Ni(NO_3_)_2_·6H_2_O, (**5k**) (NH_3_)_4_Pt(NO_3_)_2_, (**5l**) Tb(NO_3_)_3_ and (**5m**) Zn(AcO)_2_·2H_2_O. The complex precipitated immediately upon mixing the metal salt and the ligand. The complex was isolated by filtration, washed with copious amounts of water, and then dried at 60 °C in a vacuum oven. Product yields were above 90% to quantitative. Characterizations are shown in Supplementary Figs 6–8.

Amorphous melon with the cyanamide functionalization was prepared following the original synthesis of potassium melonate^38^, except that the water-insoluble solid was collected. In detail, melon (800 mg) was thoroughly ground with KSCN (1.6 g, dried at 140 °C in vacuum) and loaded in an alumina boat. In a tube furnace, this mixture was heated under argon to 400 °C at 30 °C min^−1^ ramp for 1 h, and then to 500 °C at 30 °C min^−1^ ramp for 30 min. The resulting yellow mass was suspended in water and the insoluble product was isolated by centrifugation, washed with copious amount of water and dried at 60 °C in a vacuum oven. Yield from 800 mg melon is 350–450 mg (35–45% assuming the formula C_7_N_10_H_1.4_K_0.6_, see elemental analyses in Supplementary Table 4).

**Figure 1:**
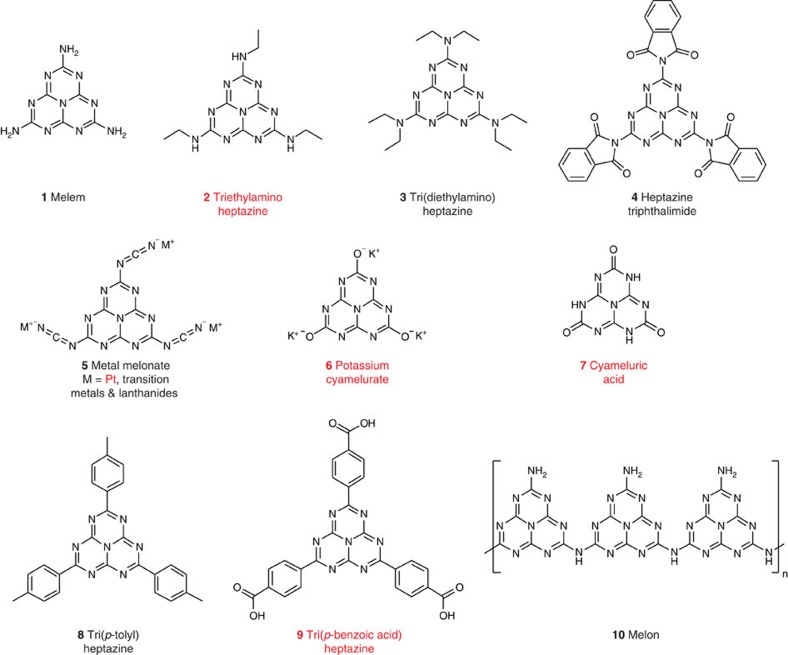
Molecular models used in this study. Molecular structures of the photocatalyst models, with the ones exhibiting photocatalytic activity for hydrogen evolution under standard conditions labelled in red. Sample codes for the melonate complexes with different metals are summarized in the Supplementary Information. Both melem and melon are denoted as inactive based on their crystalline counterparts, rather than the structurally ambiguous amorphous versions (see supporting discussion regarding structure)^18^.

**Figure 2:**
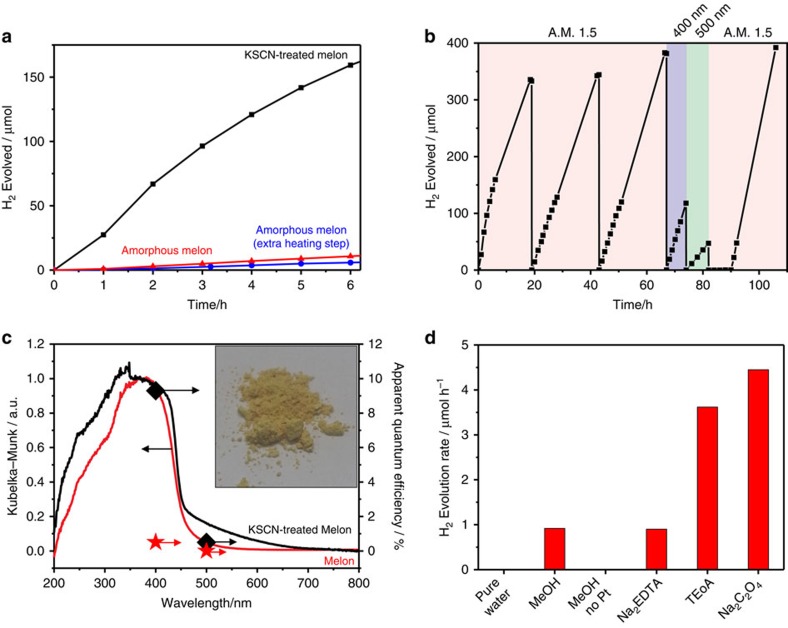
Photocatalytic evaluation of KSCN-treated melon compared with amorphous melon. (**a**) Photocatalytic hydrogen evolution of KSCN-treated melon compared with amorphous melon, and amorphous melon that has undergone an extra heating step identical to the KSCN-treated melon (400 °C for 1 h, then 500 °C for 30 min) for the first 6 h and for (**b**) 100+h. After every overnight run, the headspace of the reactor was evacuated and flushed with argon. Methanol (500 μl) was added on the 43rd and 67th hour, and on the 74th, 82nd and the 90th hours (200 μl). Note that the rate only appears to be increasing because the headspace sampling was carried out manually and, hence, at irregular intervals. See main text for the average rates. (**c**) Action spectra of KSCN-treated melon and melon showing photocatalytic activity over two wavelengths, with the black diamonds and red stars corresponding to the AQE of the KSCN-treated melon and melon, respectively; inset shows a photograph of the KSCN-treated melon. (**d**) Comparison of the hydrogen evolution rate in different electron donors, using ethylenediamine tetra-acetic acid (disodium salt, Na_2_EDTA), methanol (MeOH), triethanolamine (TEoA) and sodium oxalate at the optimized platinum loading. Reaction conditions are as follows: catalyst (20 mg) dispersed in aqueous solution of the donor (20 ml, 50 mM) and H_2_PtCl_6_ (40 μl of 8 wt% aqueous solution) under AM 1.5 irradiation. Control experiments conducted without electron donor (‘pure water') and with methanol (10 vol%), but without addition of platinum co-catalyst (‘MeOH no Pt') were also performed.

**Figure 3:**
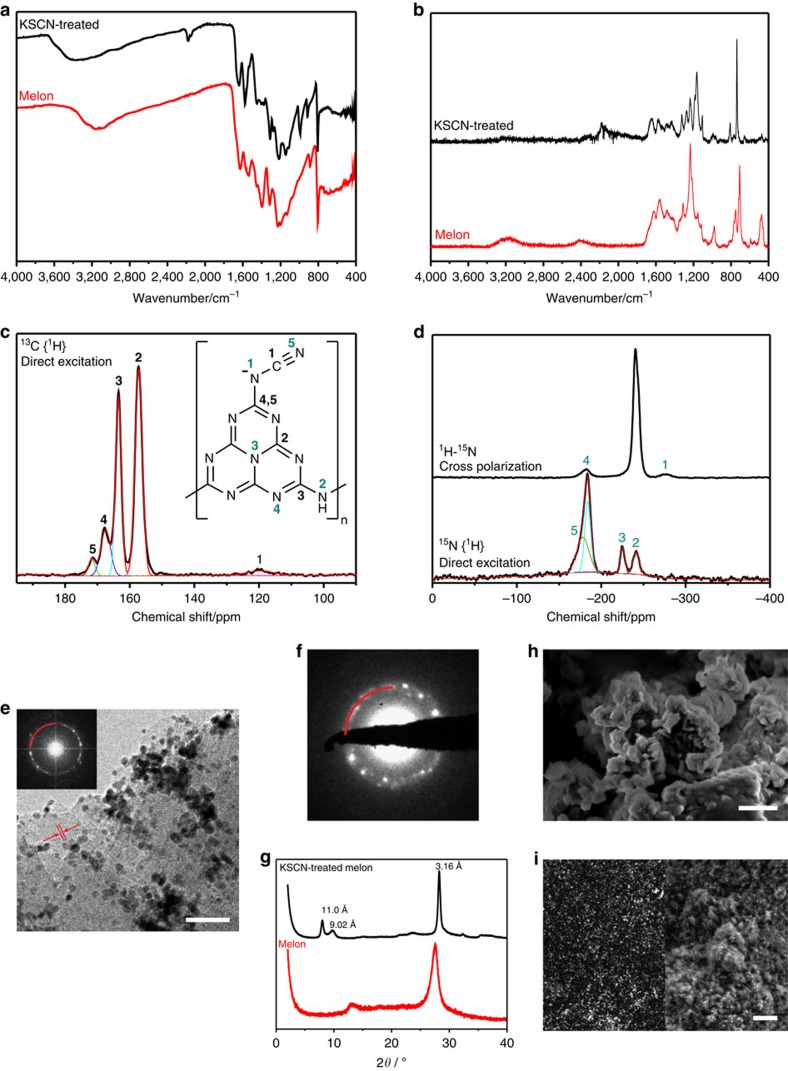
Characterizations of KSCN-treated melon compared with amorphous melon. (**a**) FTIR, (**b**) Raman, solid-state magic angle spinning (MAS) (**c**) ^13^C and (**d**) ^15^N NMR spectra of KSCN-treated melon. For the ^15^N spectrum, melon was treated in isotope-enriched KS^13^C^15^N. A summary of the deconvolution and integration of the ^13^C spectrum is given in Supplementary Table 4. Inset shows the proposed structure and NMR assignments (carbons 4 and 5 refer to H^+^ and K^+^ as counterions, respectively); (**e**) bright-field TEM image of the spent catalyst with the *in situ* deposited platinum particles of diameter 2–4 nm (scale bar, 20 nm) and the FFT of the image (inset); (**f**) selected area electron diffraction pattern of the spent catalyst; all markings in red for both **e**,**f** indicate a lattice spacing of 11 Å; (**g**) X-ray diffraction patterns; (**h**) SEM before catalysis (scale bar, 200 nm); (**i**) SEM of the spent catalyst imaged with ESB (energy and angle selective) backscattered electron (left) and imaged with secondary electron (right) detector (scale bar, 200 nm); the ESB-backscattered image shows the photoreduced platinum as bright spots.

**Figure 4:**
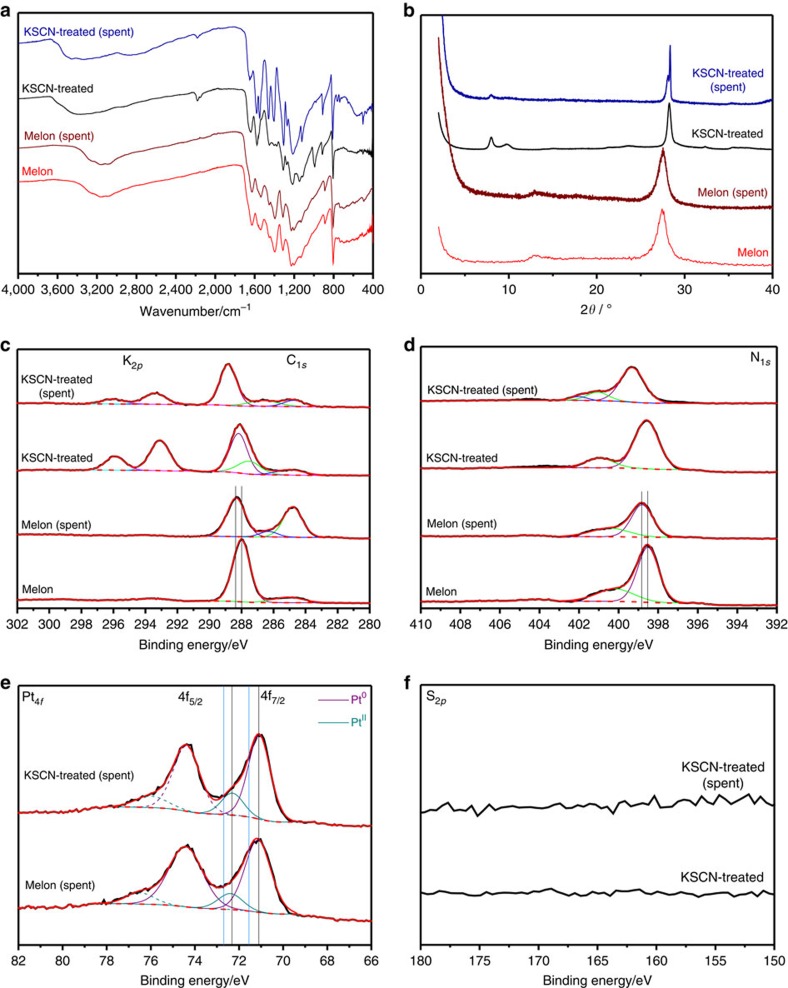
Characterization of the KSCN-treated melon before and after the 100+ h photocatalytic experiment. (**a**) FTIR, (**b**) X-ray diffraction, and XPS in the regions of (**c**) K_2*p*_ and C_1*s*_, (**d**) N_1*s*_, (**e**) Pt_4*f*_ and (**f**) S_2*p*_. Markings in the C_1*s*_ and N_1*s*_ XPS spectra indicate the slight shifts observed between the pristine and spent photocatalysts. In the Pt_4*f*_ spectrum, for comparison of the B.E. of the platinum on the melon samples, light blue lines are drawn to show the B.E. (adjusted for the different calibrations used) of Pt^0^ on graphite^70^ and Pt^2+^ on SiO_2_,^70^ both non-strongly interacting supports.

**Figure 5:**
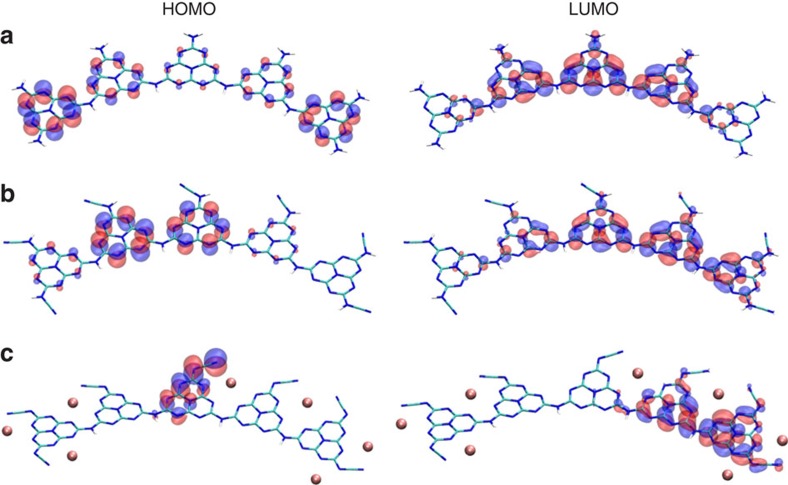
HOMO and LUMO distribution on the carbon nitrides. (**a**) Melon pentamer. (**b**) and (**c**) Melon pentamer with all -NH_2_ replaced by NCN-, charge balanced by (**b**) protons and (**c**) potassium, calculated using the ic-PBEh functional (see page S8 and S36 for computational details).

As post-synthetic annealing may lead to a significant improvement in the photocatalytic activity of melon, we prepared another control sample to verify that the large outperformance of KSCN-treated melon is not attributed to this heating step. Melon in a ceramic boat was heated under argon to 400 °C at 30 °C min^−1^ ramp for 1 h, and then 500 °C at 30 °C min^−1^ ramp for 30 min. This sample is denoted as ‘amorphous melon (extra heating step)' in Fig. 1a.

### Analytical methods

Liquid-state ^1^H, ^13^C NMR spectra were collected on a Bruker Avance 300 MHz NMR spectrometer at resonance frequencies of 300 and 75.46 MHz, respectively (*B*_0_=7.04 T). X-ray diffraction patterns were collected using a STOE Stadi P diffractometer (Cu Kα1) in the transmission mode. ATR-IR spectra were collected with a PerkinElmer UATR TWO spectrometer equipped with a diamond crystal. Raman spectra were recorded with a Bruker MultiRAMII FT-Raman spectrometer with Nd:YAG laser excitation (1,064 nm) at power up to 1,000 mW at range between 400 and 4,000 cm^−1^ with 1 cm^−1^ resolution; samples were loaded in a glass tube for measurement. Diffuse reflectance UV–Vis spectra were collected on a Cary 5,000 spectrometer (referenced to polytetrafluoroethylene (PTFE) or barium sulfate) and the spectra in percentage reflectance were converted using the Kubelka Munk function. CHN elemental analyses were performed with a Vario El element analyser (Elementar Analysensysteme GmbH). ICP-AES elemental analyses were carried out using a VISTA RL CCD and ICP-AES analyser system (Agilent Technologies, Waldbronn). Sorption measurements were performed on a Quantachrome Autosorb iQ gas sorption analyser using argon as the sorbent at 87.45 K. Samples were outgassed overnight at 150 °C to a vacuum of 10^−7 ^mbar. Surface areas were calculated using the BET theory from the argon adsorption isotherms of the samples. Pore size distribution and volume were calculated from the adsorption isotherm employing either the non-local or quenched solid DFT using the ‘Ar-Carbon cylindrical pores at 87 K' or the ‘Ar-Carbon slit pores at 87 K' kernel (applicable pore diameters 0.35–36 nm) as implemented in the AUTOSORB data reduction software. TEM was performed with a Philips CM30 ST (300 kV, LaB6 cathode) and the images were recorded with a CMOS Camera (TemCam-F216, TVIPS). The samples were suspended in n-butanol and drop-cast on a lacey carbon film (Plano). SEM was performed on a Zeiss Merlin electron microscope. For XPS, samples were pressed on indium foil and the spectra were collected on an Axis Ultra (Kratos Analytical, Manchester) X-ray photoelectron spectrometer with charge neutralization. The spectra were processed using the software CasaXPS 2.3.16. The spectra were referenced with the adventitious carbon 1*s* peak at 284.80 eV. Binding energies were compared with the NIST Standard Reference Database 30 (Version 4.1, http://srdata.nist.gov/xps/Default.aspx) unless otherwise specified. TGA-MS was performed with the instrument STA 409 C (Netzsch GmbH, Selb, Germany) connected with a quadrupole mass spectrometer QMS 422 (Balzers, Hudson, USA). Samples were loaded in alumina crucibles and heated under argon (100 ml min^−1^) from ambient temperature to 900 °C at a ramp rate of 1 °C min^−1^.

Solid-state magic angle spinning NMR measurements, photocatalytic experiments and DFT calculations are described in detail in the Supplementary Methods.

### Data availability

The data that support the findings of this study are available from the corresponding author upon request.

## Additional information

**How to cite this article:** Lau, V. W.-h. *et al*. Rational design of carbon nitride photocatalysts by identification of cyanamide defects as catalytically relevant sites. *Nat. Commun.* 7:12165 doi: 10.1038/ncomms12165 (2016).

## Supplementary Material

Supplementary Information Supplementary Figures 1-27, Supplementary Tables 1-7, Supplementary Discussion, Supplementary Methods and Supplementary References.
