# Supplementary material for: Rational design of carbon nitride photocatalysts by identification of cyanamide defects as catalytically relevant sites
Source: Nat Commun. 2016 Jul 8;7:12165. doi: 10.1038/ncomms12165 (PMC4941108; doi:10.1038/ncomms12165)
Supplement: Supplementary Information — Supplementary Figures 1-27, Supplementary Tables 1-7, Supplementary Discussion, Supplementary Methods and Supplementary References. [file ncomms12165-s1.pdf]

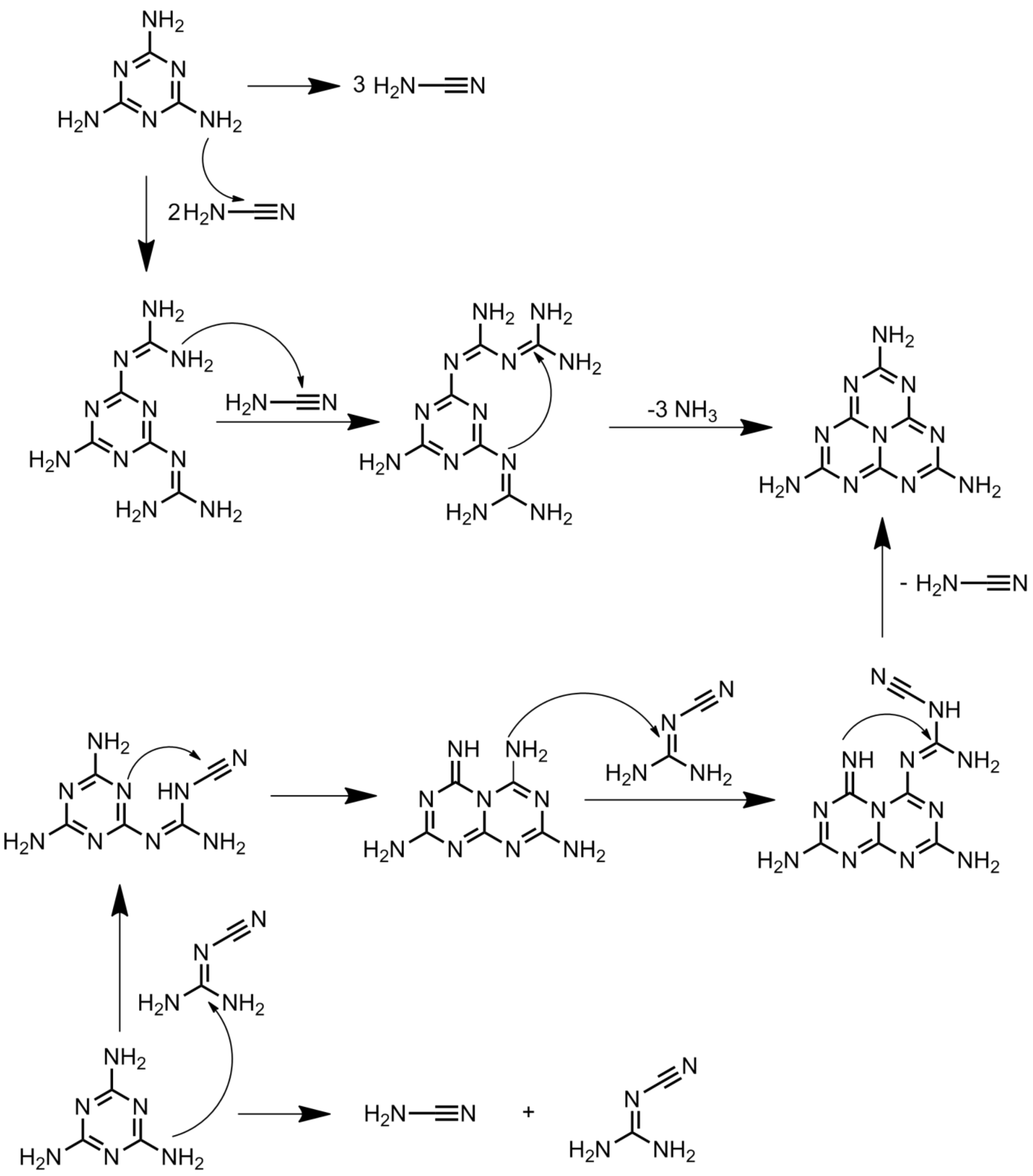

Supplementary Figure 1: Proposed mechanisms of hepazine formation from melamine, adapted from Schwarzer *et al.*[1] The top mechanism is suggested by May,[2] while the bottom one is collated from the works of Hosmane *et al.*[3,4]

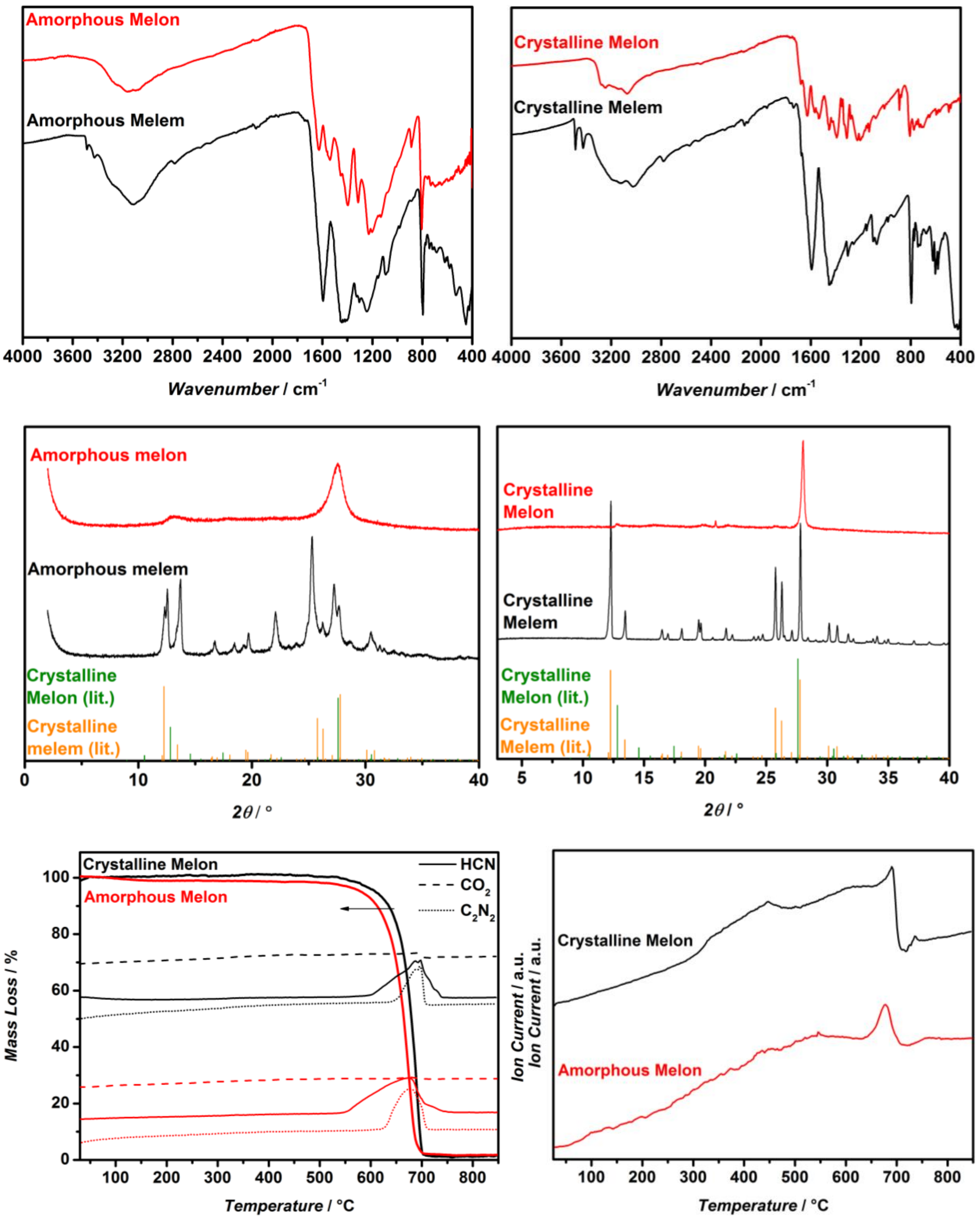

Supplementary Figure 2: FTIR comparison of amorphous (top left) and crystalline (top right) melon and melem, XRD comparison of amorphous (middle left) and crystalline (middle right) melon and melem; TGA-MS (bottom left) of crystalline and amorphous melon, with the CO_2 evolution shown in bottom right.

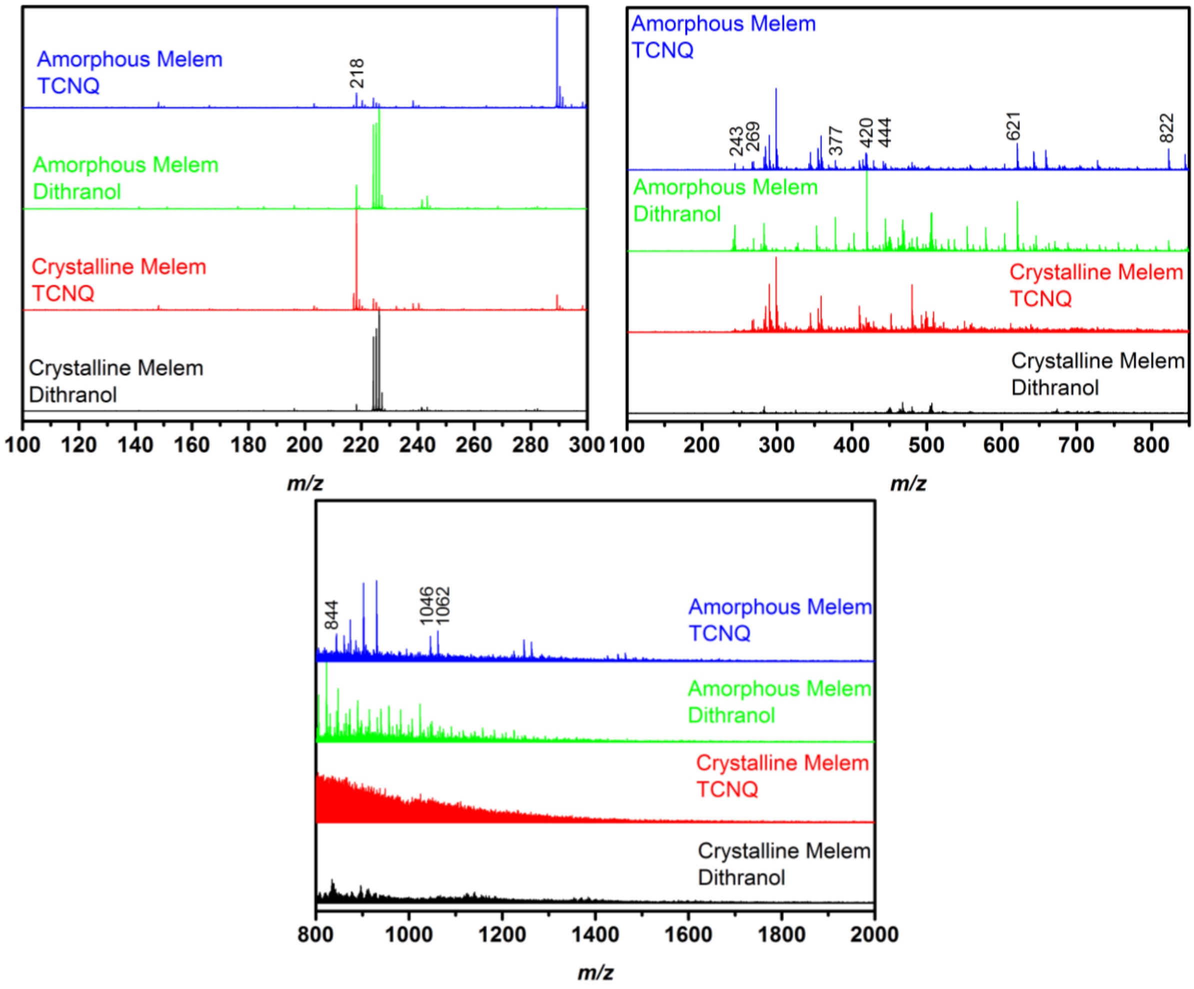

Supplementary Figure 3: MALDI-TOF spectra of crystalline and amorphous melem at the >100 (top left), >300 (top right) and the >800 (bottom) mass range.

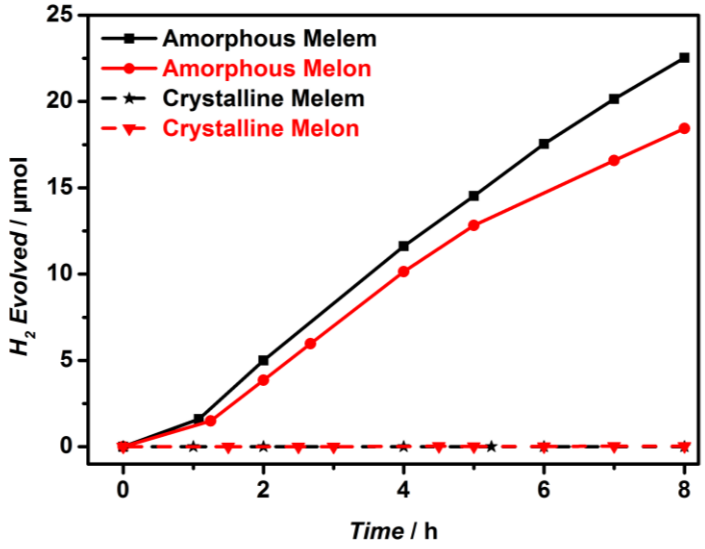

Supplementary Figure 4: Photocatalytic activity of amorphous and crystalline melem and melon. Photocatalytic reactions were conducted under irradiation from the full spectrum of xenon lamp.

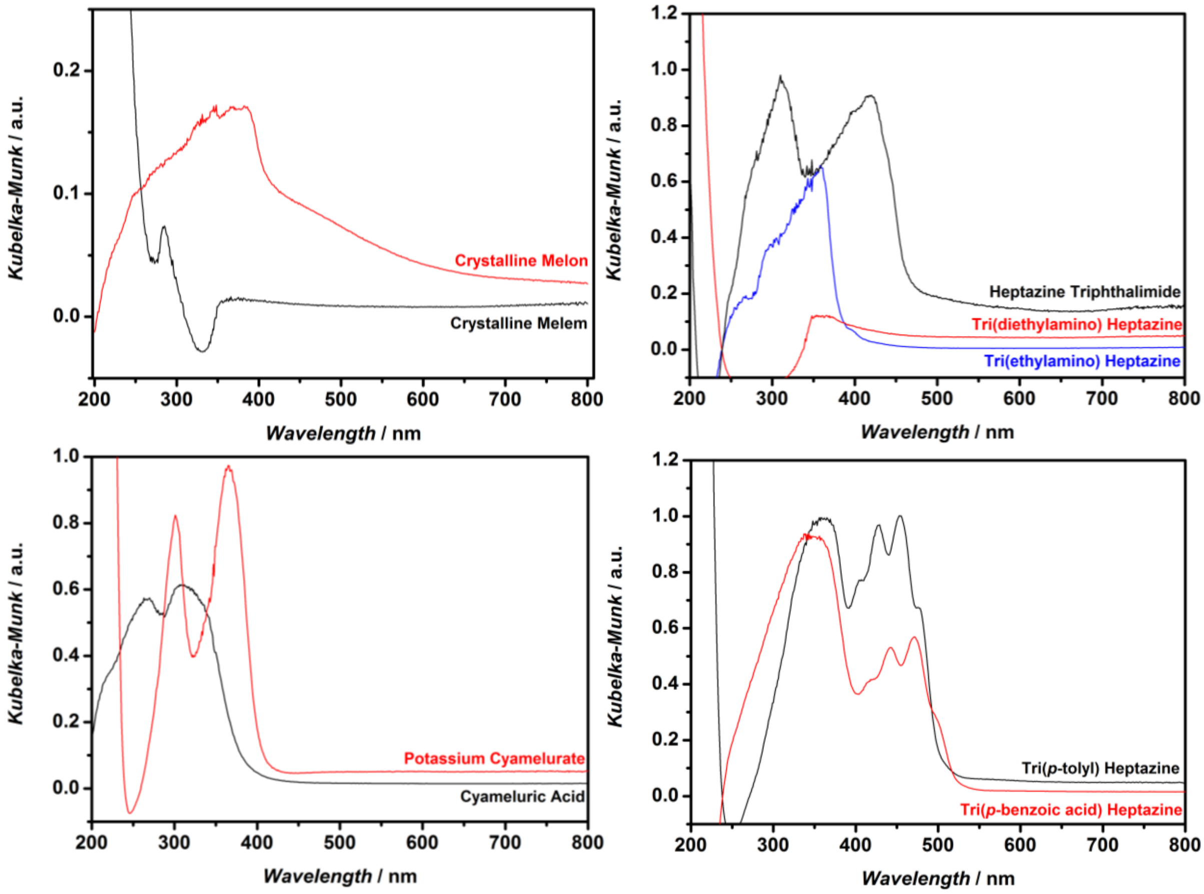

Supplementary Figure 5: Diffuse reflectance spectra of the model compounds.

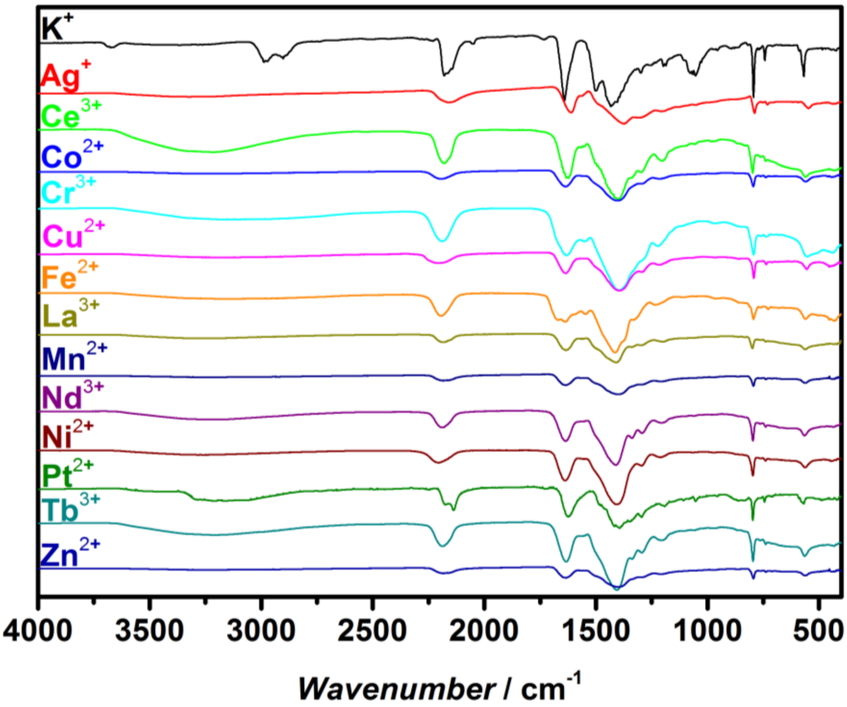

Supplementary Figure 6: FTIR of the metal melonate complexes.

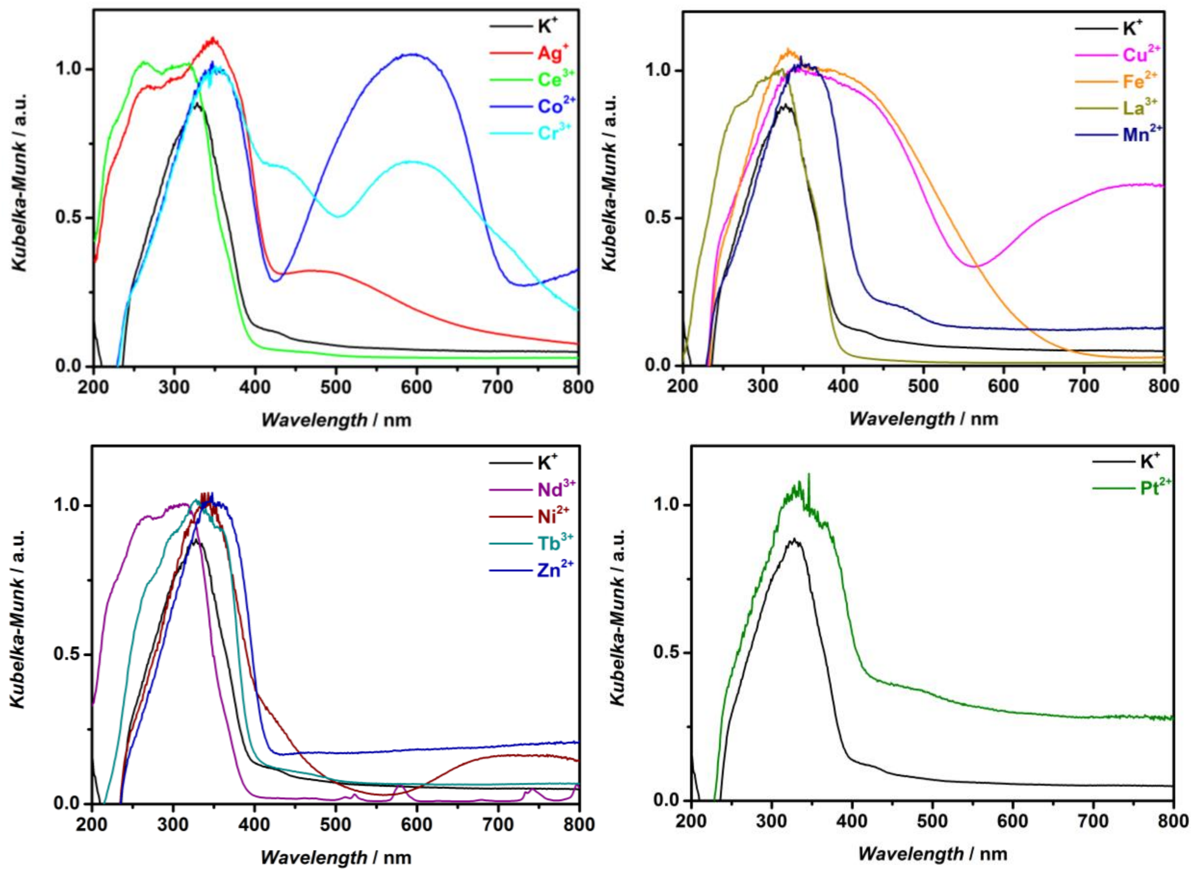

Supplementary Figure 7: Diffuse reflectance UV-Vis spectra of the metal melonates, all compared with the potassium salt.

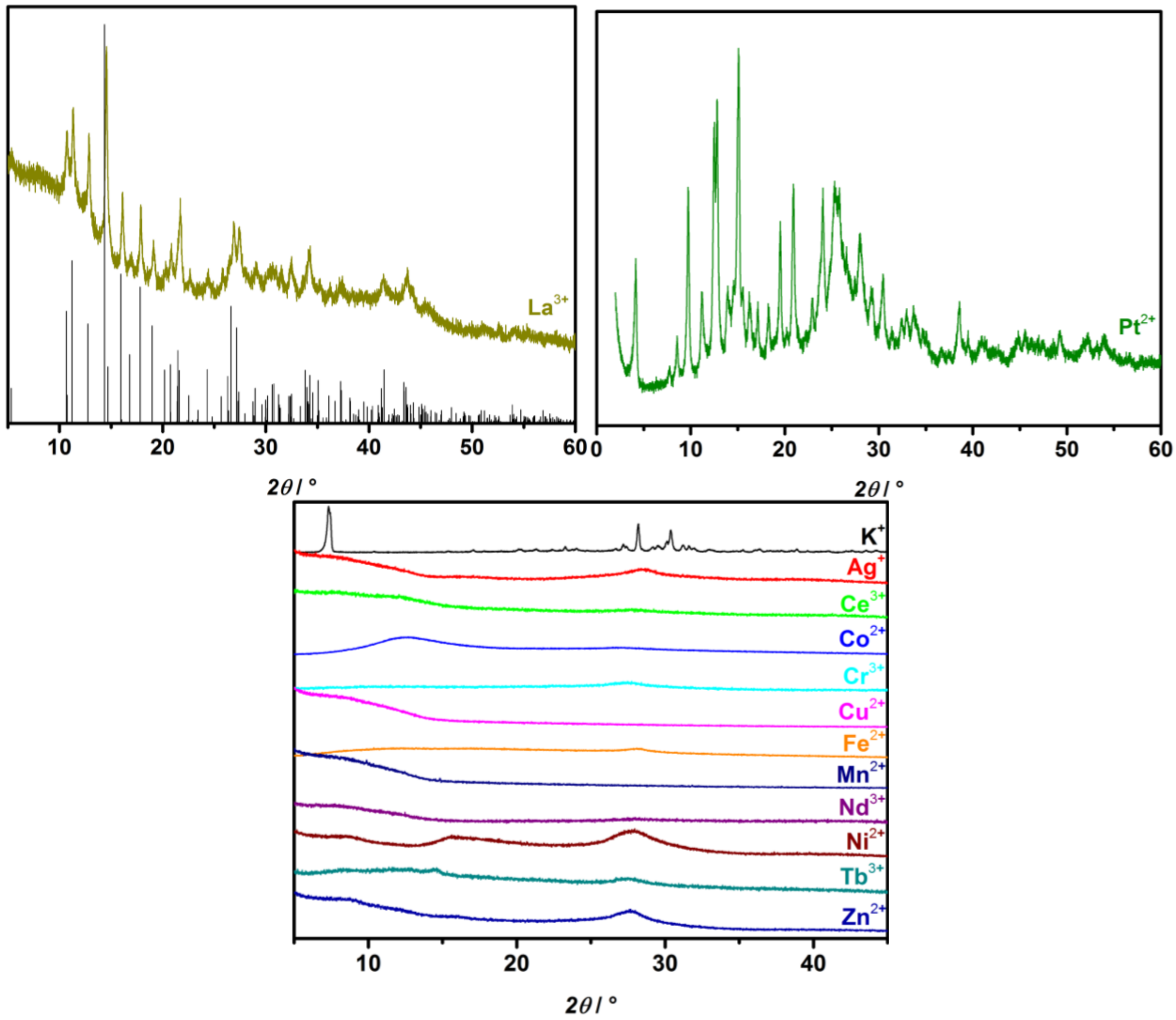

Supplementary Figure 8: XRD patterns of lanthanum melonate with a literature pattern[5] (top left), platinum melonate (top right), and the remaining melonate complexes.

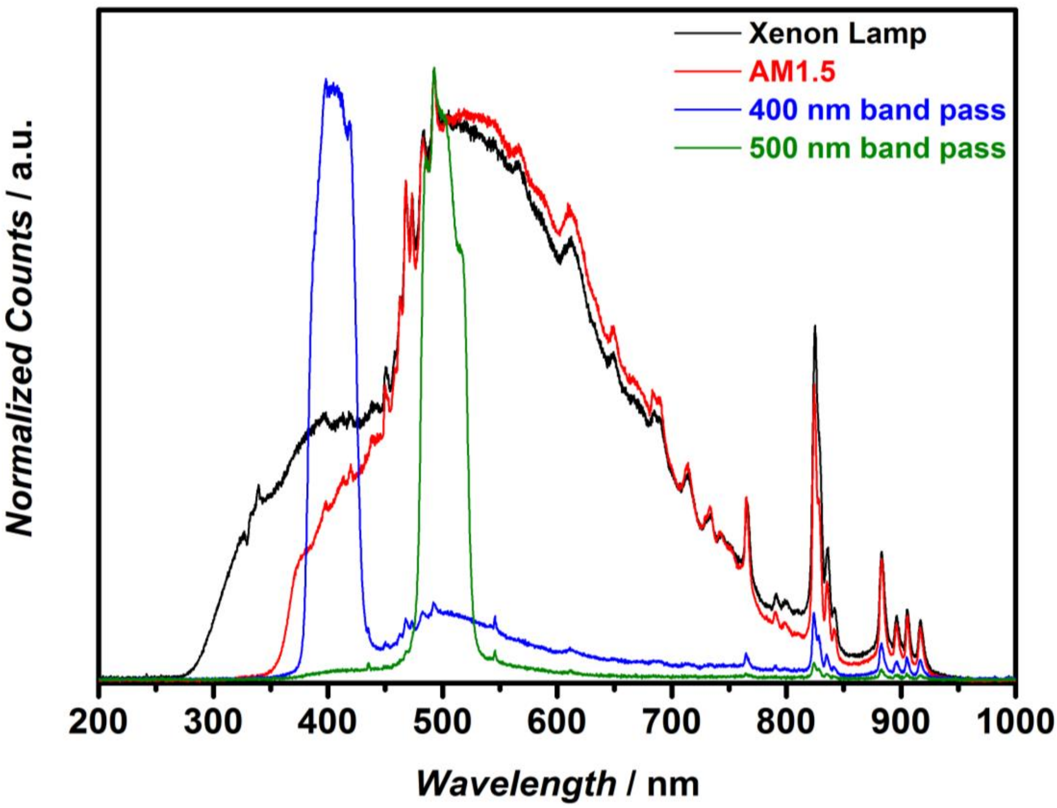

Supplementary Figure 9: Spectral profile of the irradiation source and filters used in the photocatalytic experiments.

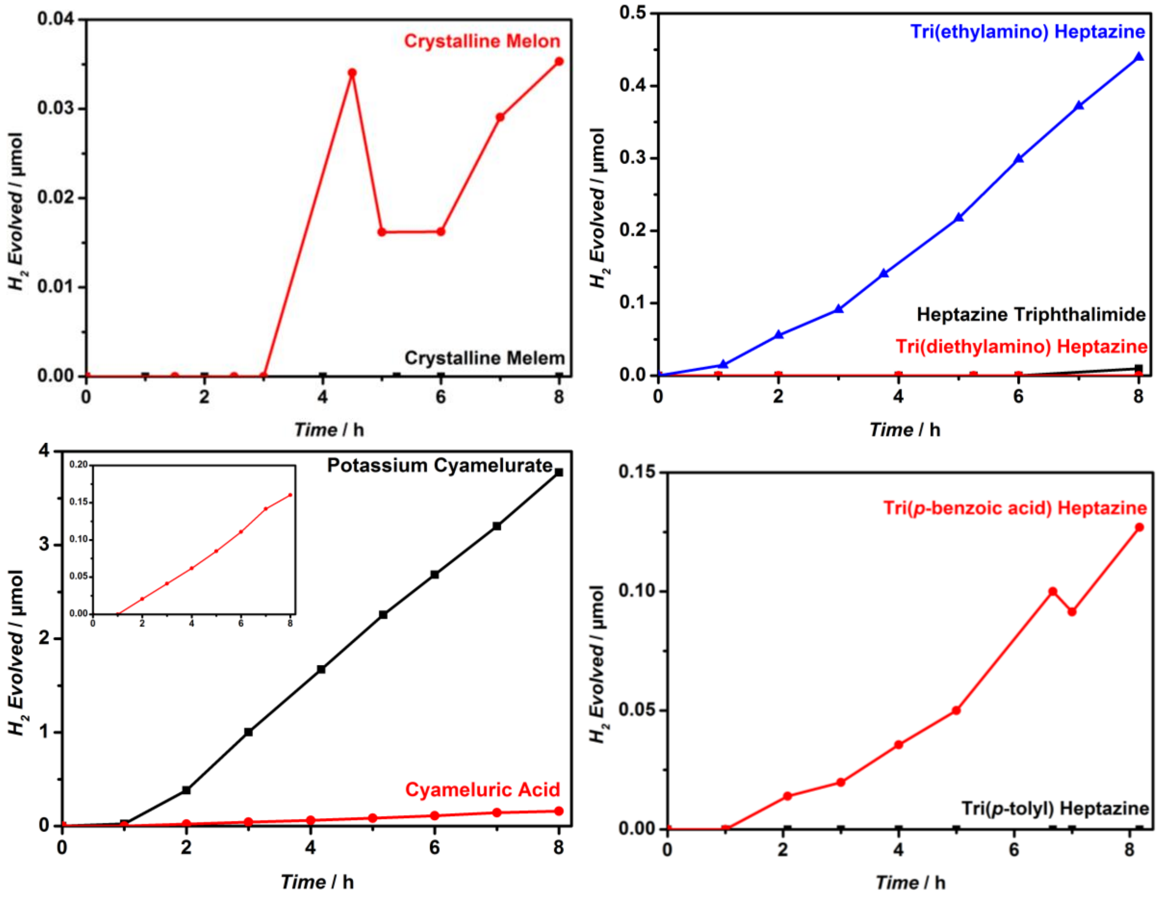

Supplementary Figure 10: Photocatalytic hydrogen evolution of the model compounds under screening conditions: the catalyst (20 mg) was dispersed in aqueous methanol (10 vol%, 20 mL) and H_2PtCl_6 (5 µL of 8 wt% aqueous solution) irradiated under full spectrum of xenon lamp. Inset of bottom left is an enlarged plot of the photocatalytic hydrogen evolution rate of cyameluric acid. Note that the hydrogen evolved for crystalline melon (top left) appears erratic because the amount evolved is at the detection limit of the GC and the process of sampling already changed the concentration significantly.

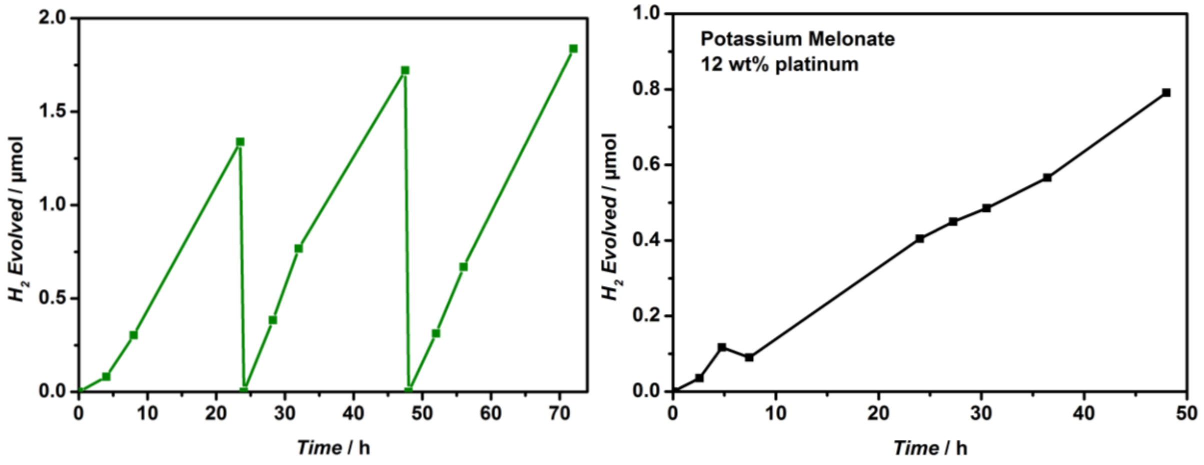

Supplementary Figure 11: Photocatalytic activity under the standard screening protocol of platinum melonate (left), except without the addition of H_2PtCl_6, and potassium melonate (right) with 12 times the H_2PtCl_6 than the standard protocol.

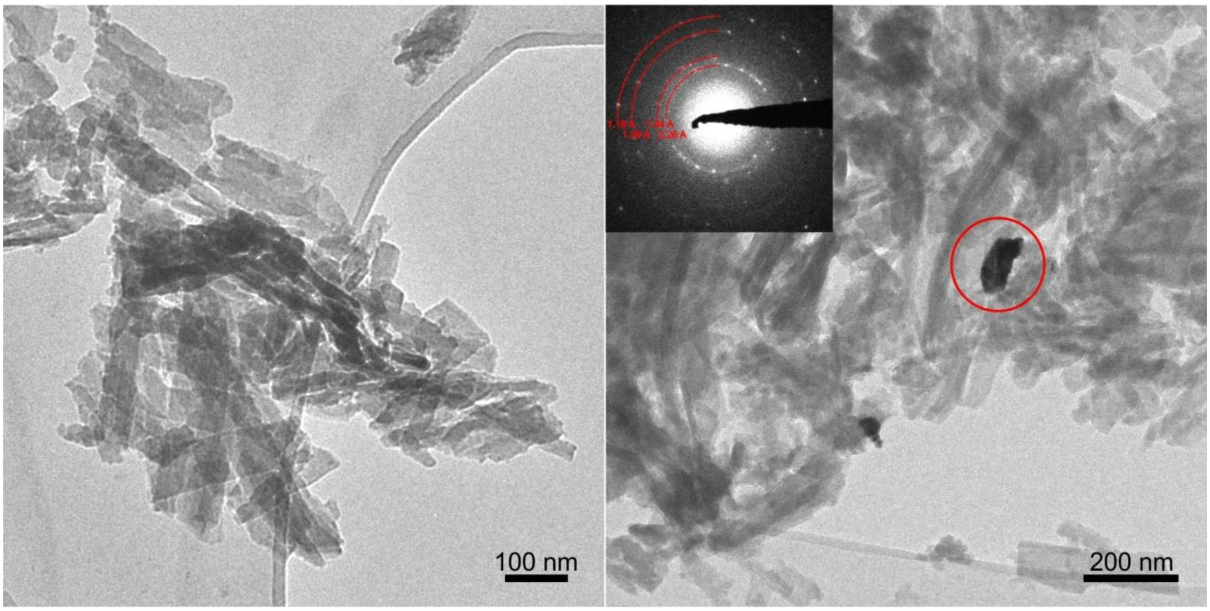

Supplementary Figure 12: Bright field TEM image of platinum melonate after the photocatalytic reaction. Pt(0) particles are not present in the majority of the sample (left) but are observed infrequently (right). Right inset shows the SAED pattern of the indicated platinum particle; the polycrystalline pattern matches that of cubic platinum(0).

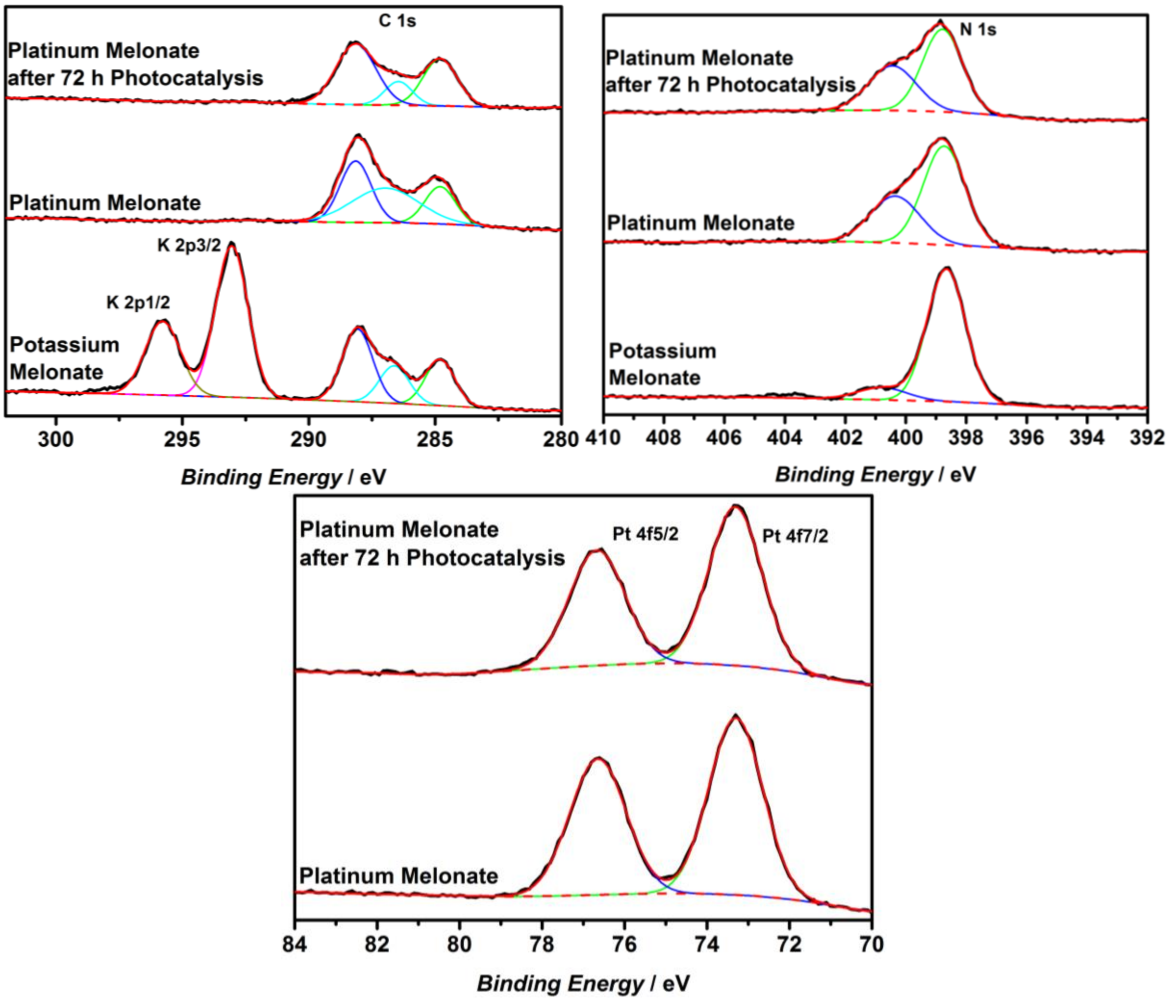

Supplementary Figure 13: XPS spectra of platinum melonate, before and after photocatalytic hydrogen evolution and compared with potassium melonate, in carbon 1s region (top left), nitrogen 1s (top right) and platinum 4f (bottom). The Pt 4f7/2 signals for the platinum melonate before and after photocatalysis is at a binding energy of 73.3 eV, which is consistent with Pt^{II} based on the NIST XPS library.

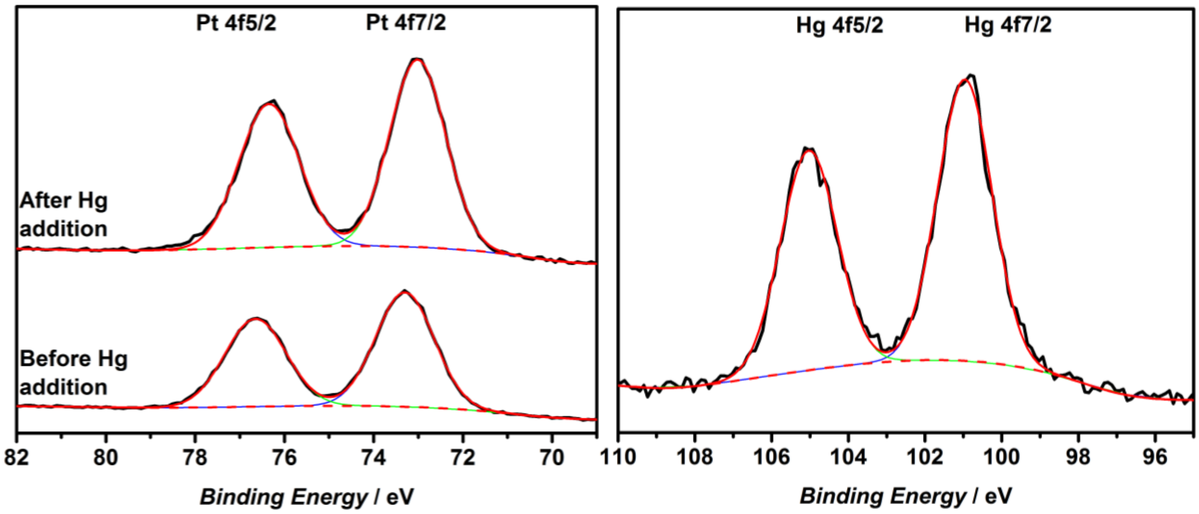

Supplementary Figure 14: XPS spectra of platinum melonate after photocatalytic reaction in the presence of elemental mercury, following the protocol for Finke's test.

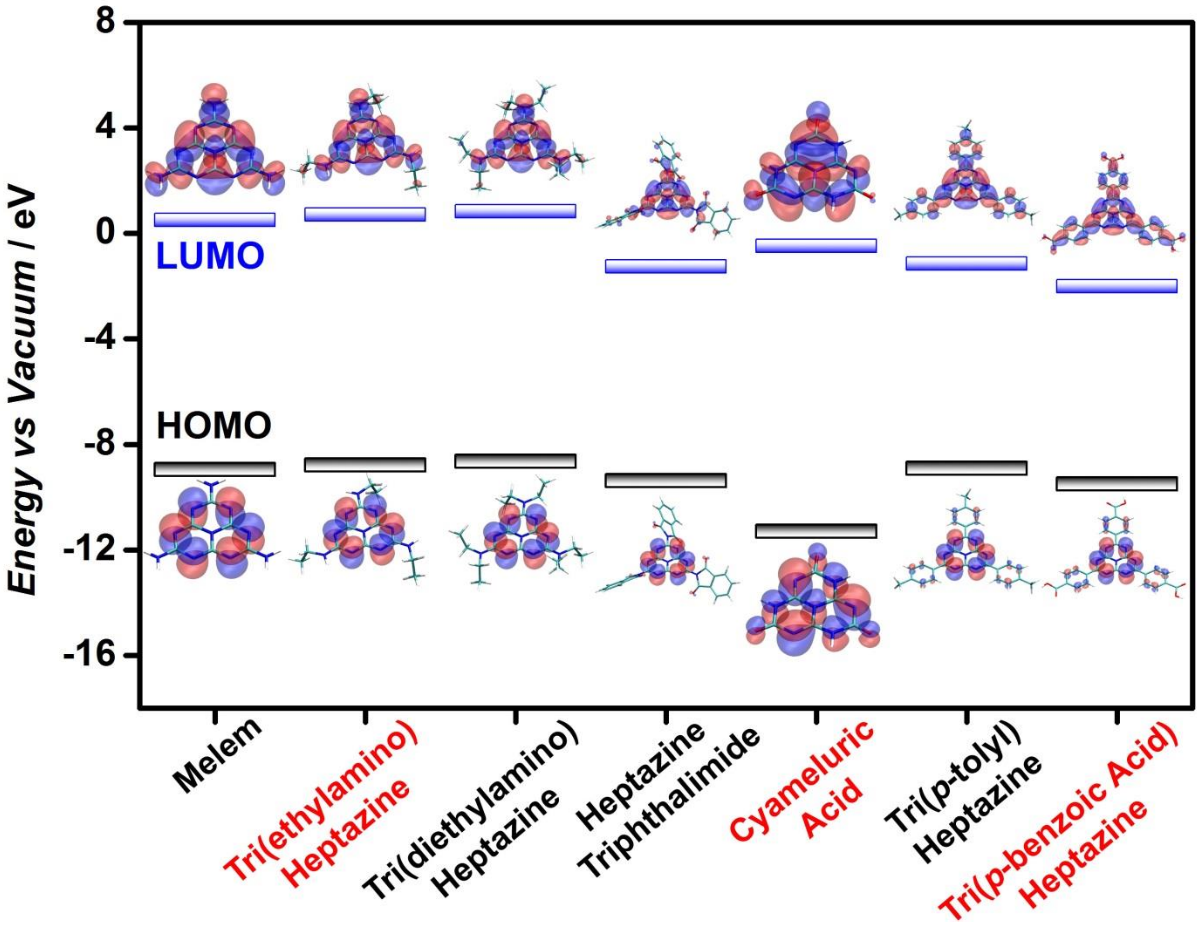

Supplementary Figure 15: Calculated frontier orbitals and energy levels of the heptazine model photocatalysts, using the ic-PBEh functional as described in the main text. The models exhibiting activity are labelled red. The models containing metal cations are not shown, since the orbital positions and energy levels can vary strongly depending on the position of the cation.

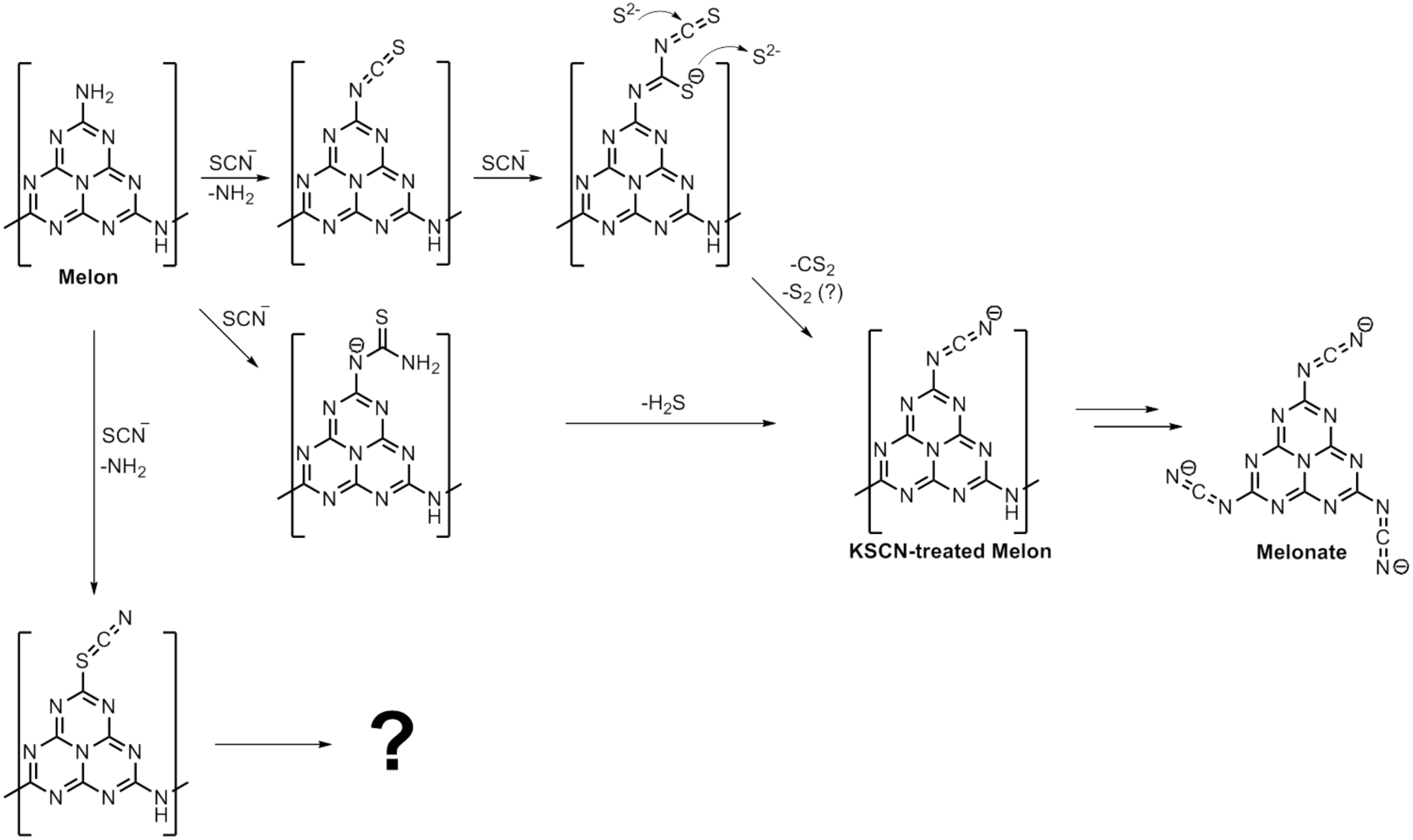

Supplementary Figure 16: Mechanisms for the formation of potassium melonate, proposed by Sattler and Schnick.[1,6]

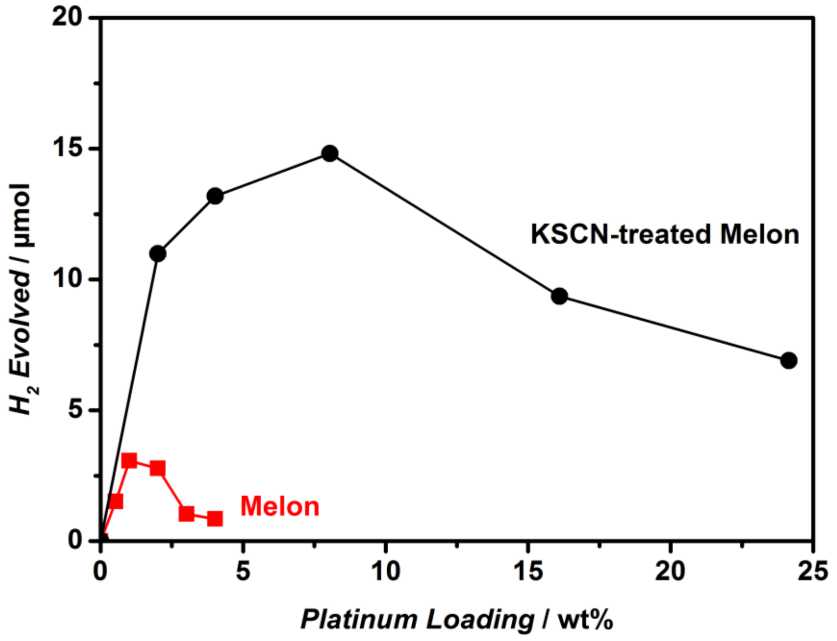

Supplementary Figure 17: Optimisation of platinum loading for hydrogen evolution.

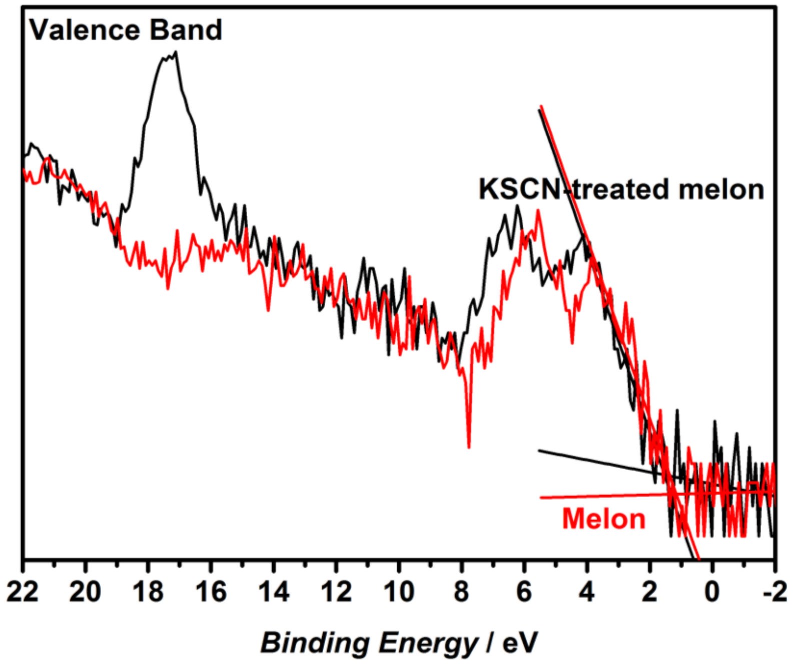

Supplementary Figure 18: XPS of the KSCN-treated melon and amorphous melon in the valence band region. Note that valence band energies were not determined by techniques such as photocurrent and Mott-Schottky plot due to the negligible conductivity of carbon nitride materials.

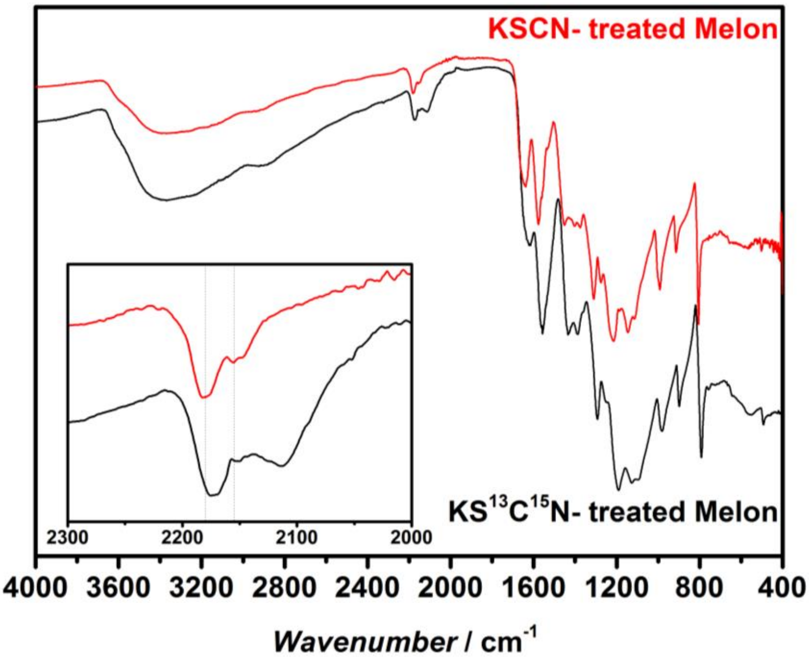

Supplementary Figure 19: FTIR of melon treated with KSCN and enriched KS^{13}C^{15}N (99% enriched for both carbon and nitrogen). Inset is an enlargement of the cyanamide region, showing the isotopic shift towards smaller wavenumbers.

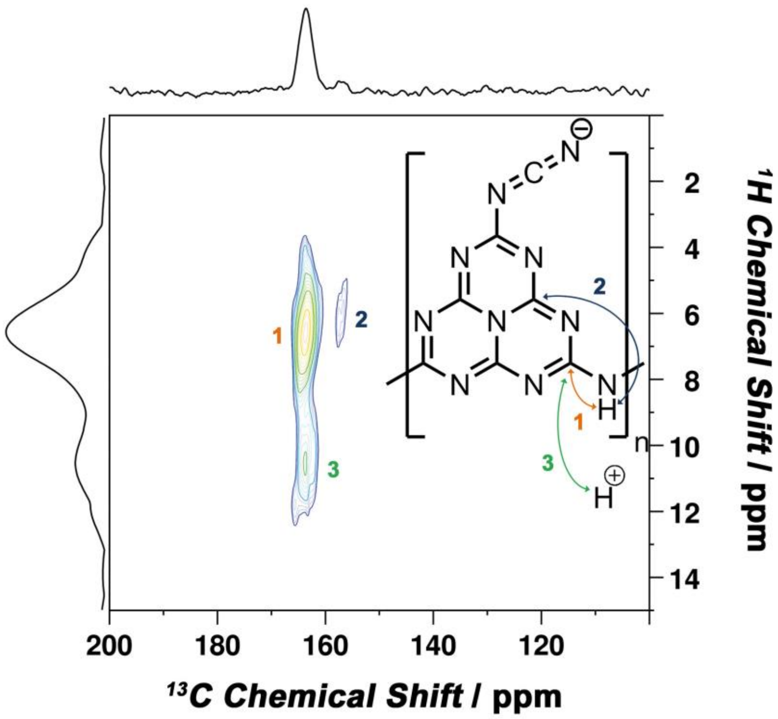

Supplementary Figure 20: Solid state ^{1}H-^{13}C FSLG HETCOR NMR spectrum of KSCN-treated melon. The sample was dried at 125 °C in vacuum over the weekend prior to measurement, due to the large water content observed from the TGA-MS.

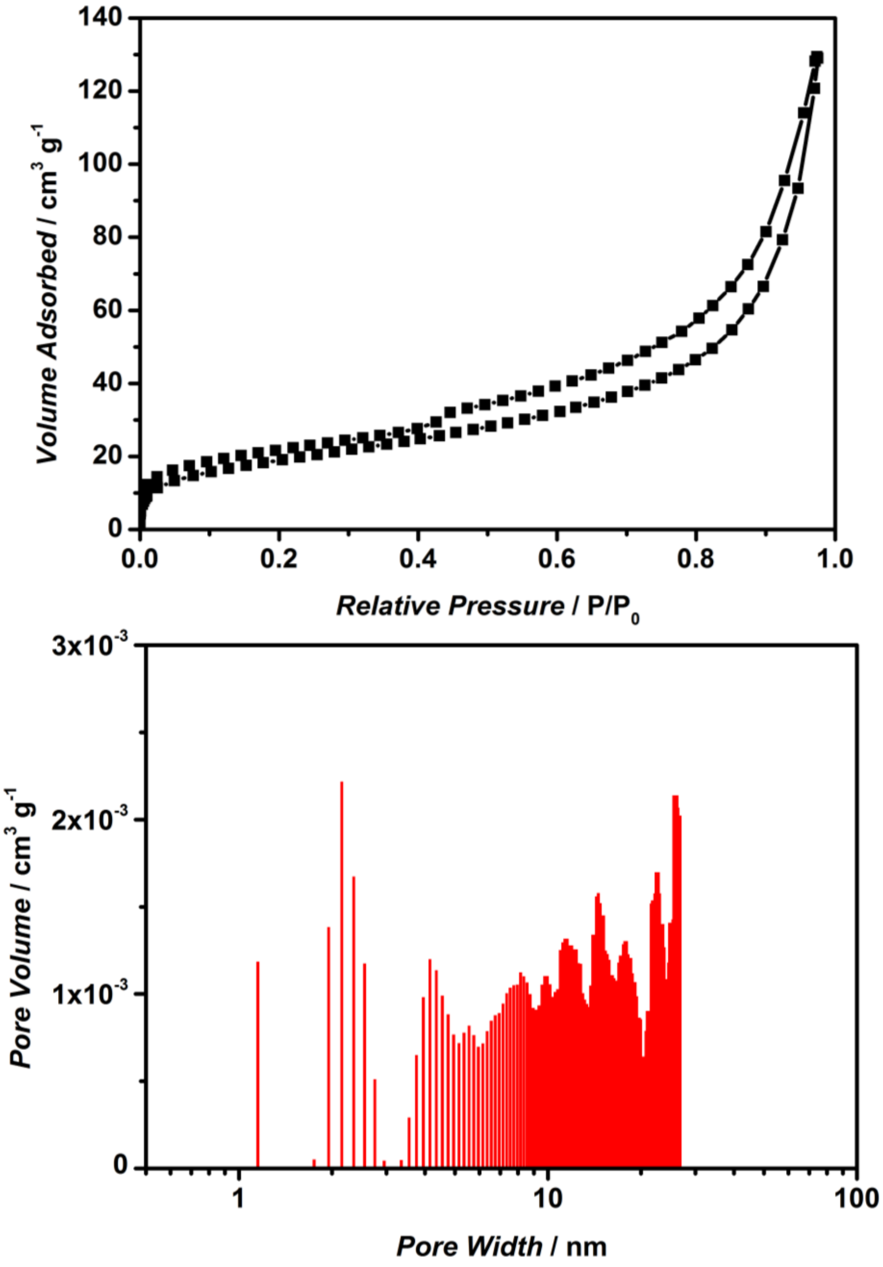

Supplementary Figure 21: Argon sorption isotherm of the KSCN-treated melon (top) and pore size distribution histograms of the KSCN-treated melon (bottom) using the QSDFT calculation model for cylindrical pores.

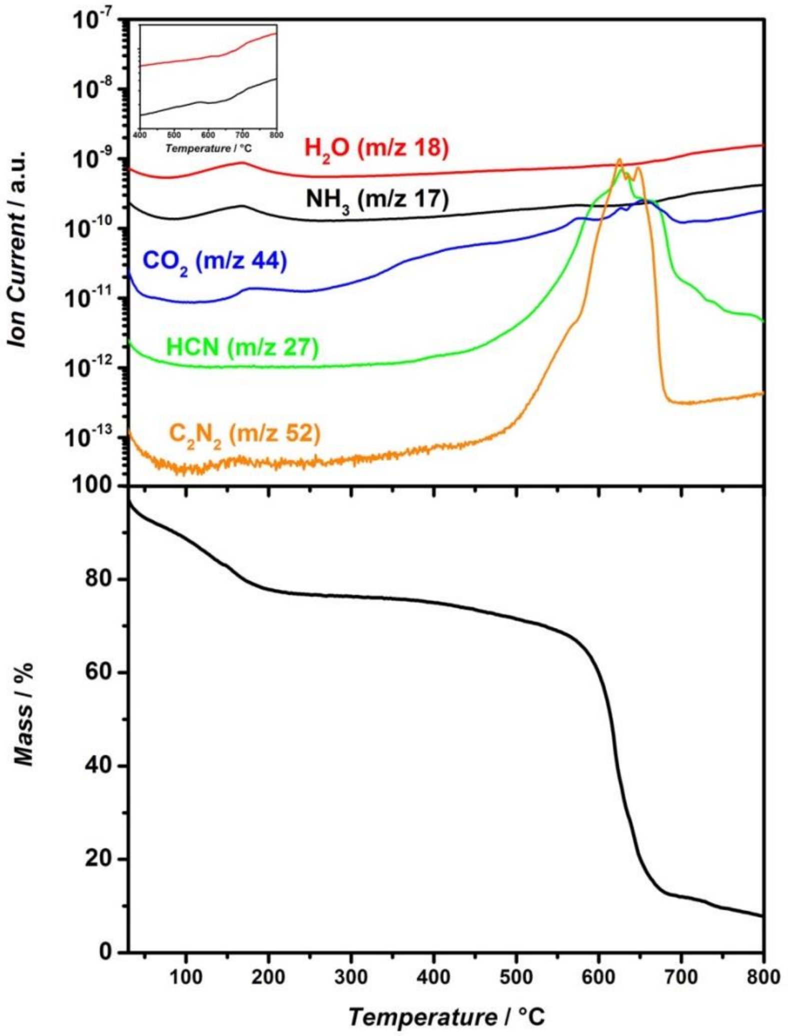

Supplementary Figure 22: TGA-MS of KSCN-treated melon. Inset shows an enlarged plot of the 400–500 °C region for *m/z* 17 and 18. Note that *m/z= 17* has been notated as NH_3, even though this can also be attributed to OH. The small peak at 575 °C observable for *m/z* 17 but not 18 (see inset) is indicative of ammonia evolution, rather than hydroxyl ion or radical.

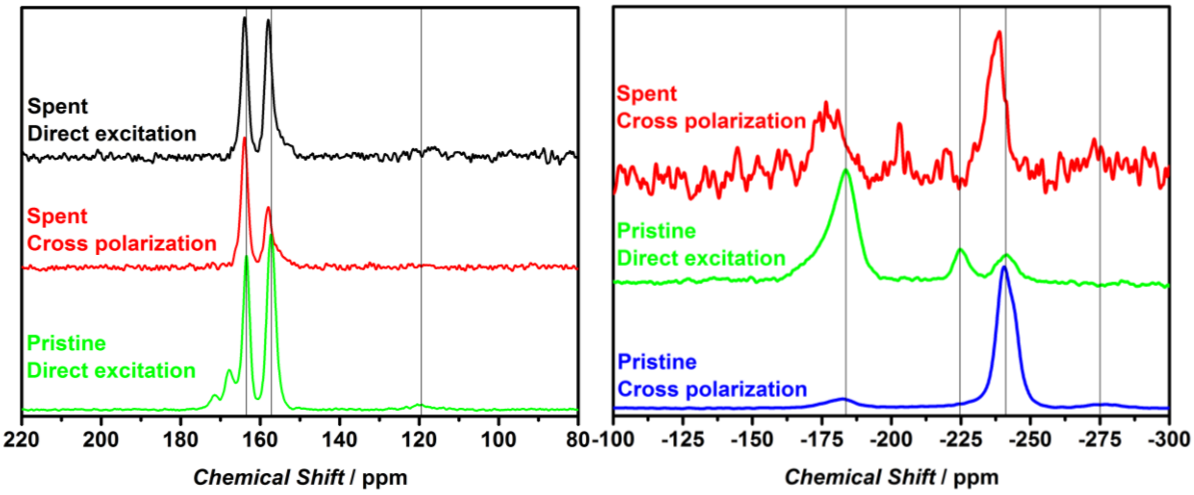

Supplementary Figure 23: ^{13}C (left) and ^{15}N (right) MAS ssNMR spectra of the KSCN-treated melon before and after 100+ h of photocatalytic reaction.

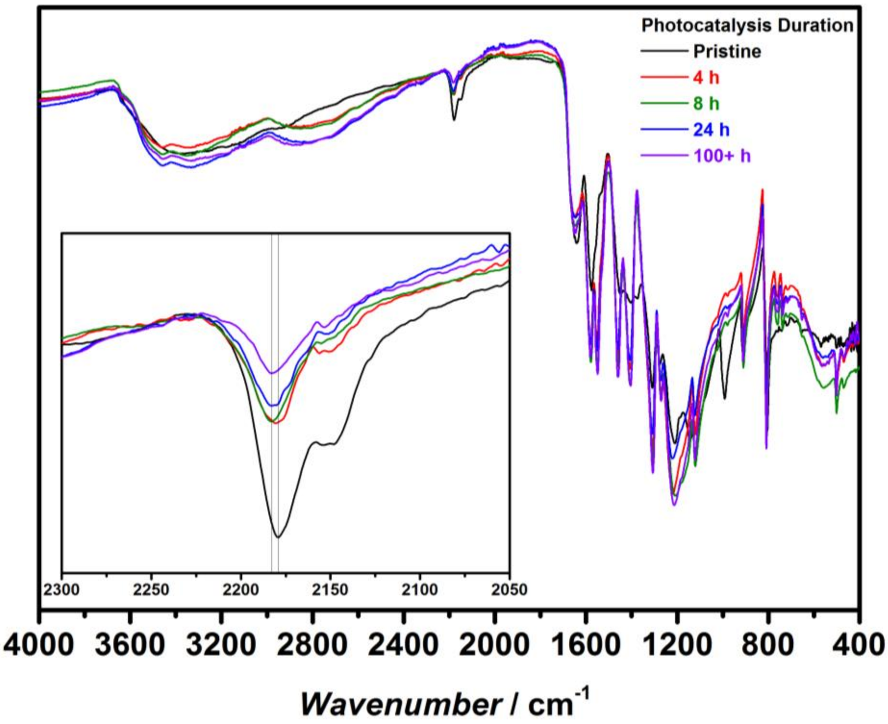

Supplementary Figure 24: FTIR of the KSCN-treated melon, collected and washed after the specified duration of the photocatalytic reaction. Inset shows the cyanamide region magnified. The intensities of all spectra have been normalized such that the heptazine signals at 809 cm^{-1} have the same intensity.

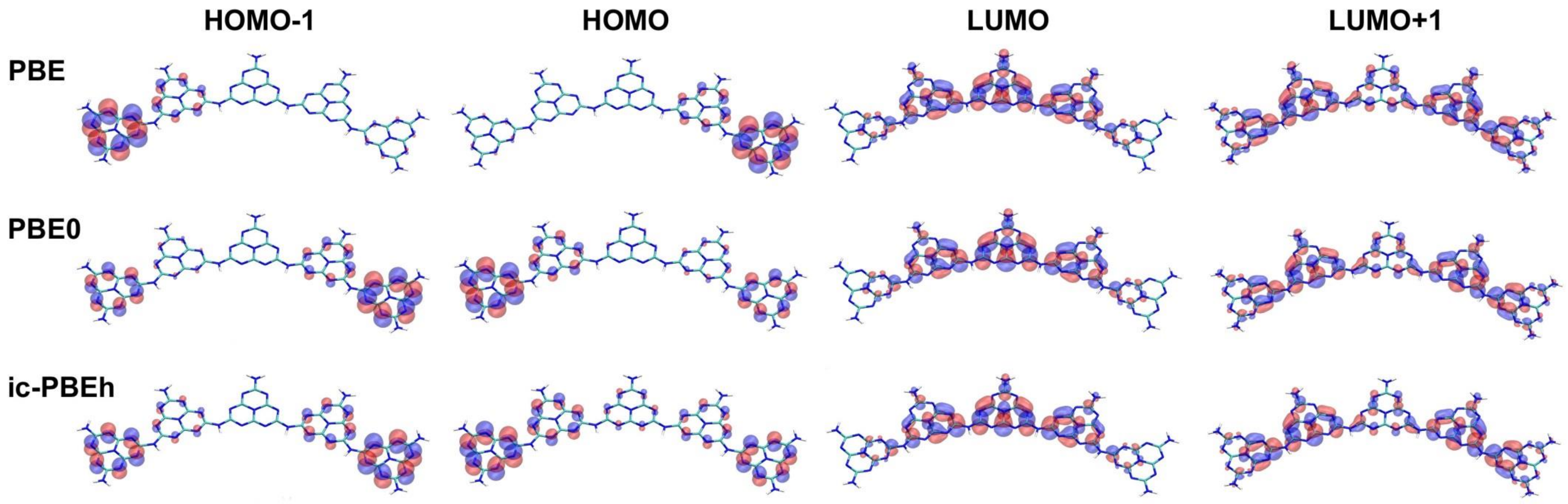

Supplementary Figure 25: HOMO and LUMO distribution of the melon pentamer, calculated using 3 different functionals. HOMO-1 refer to the case when one electron is extracted from the orbitals, while LUMO+1 is the case when the orbital is populated by one electron.

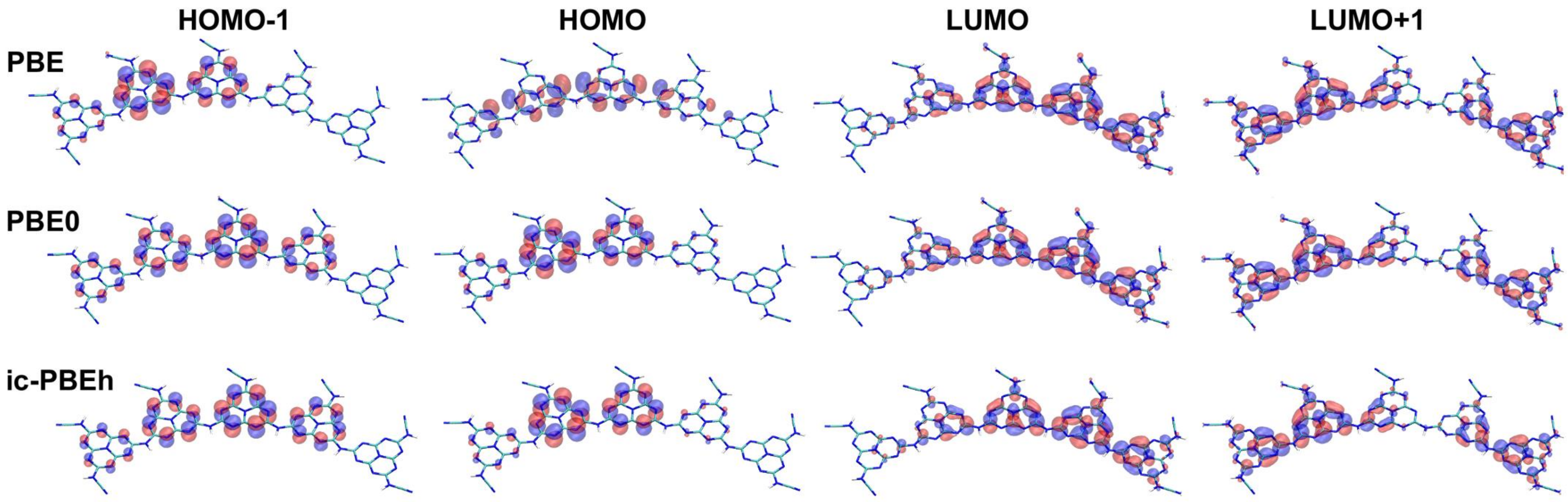

Supplementary Figure 26: Calculated HOMO and LUMO distribution of the melon pentamer with all $–NH_2$ substituted for the cyanamide $–NCN^-$ charge balanced by protons.

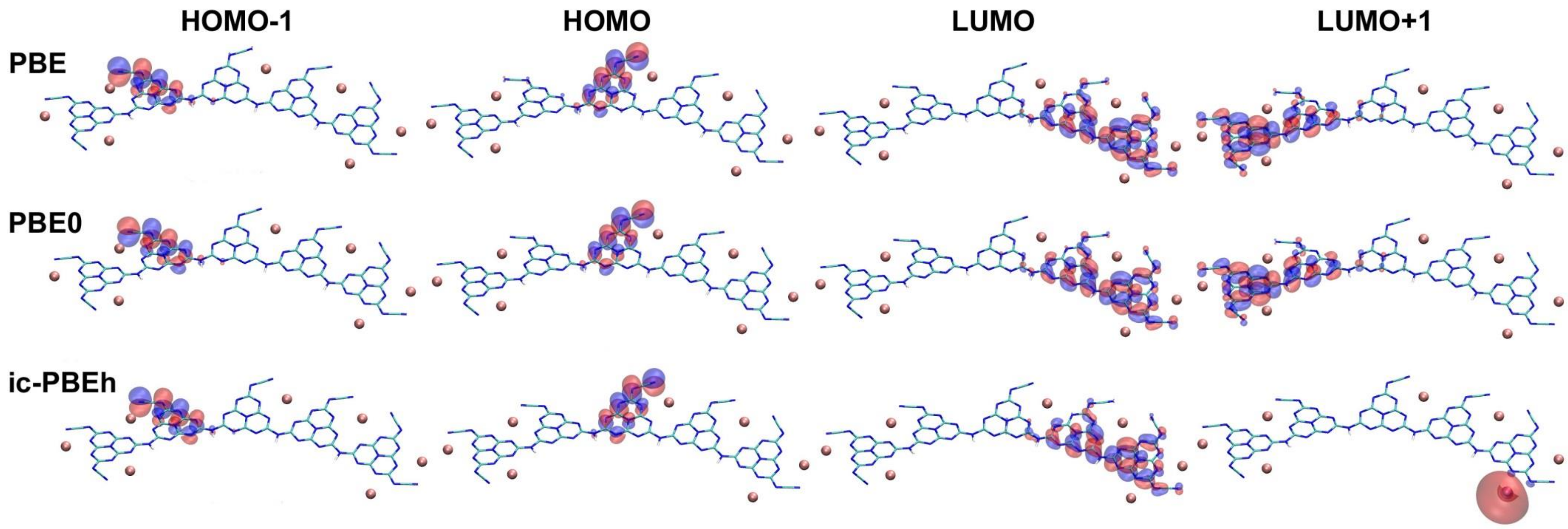

Supplementary Figure 27: Same as Supplementary Figure **26**, except the model used has potassium cation as the charge balance.

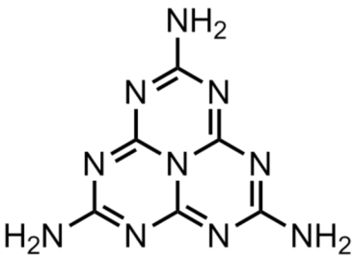

218

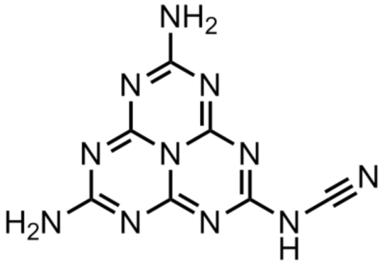

243

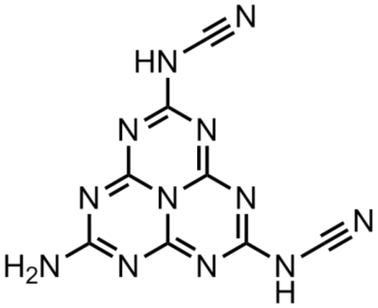

268

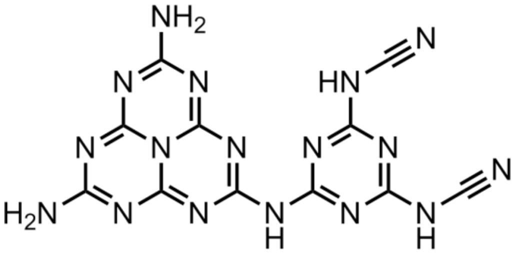

377

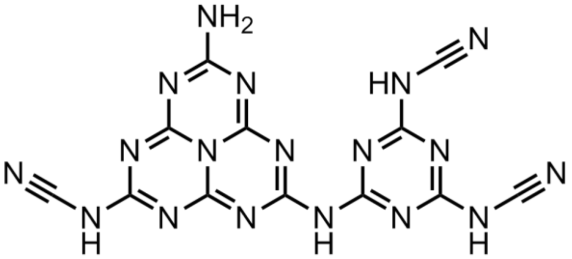

402

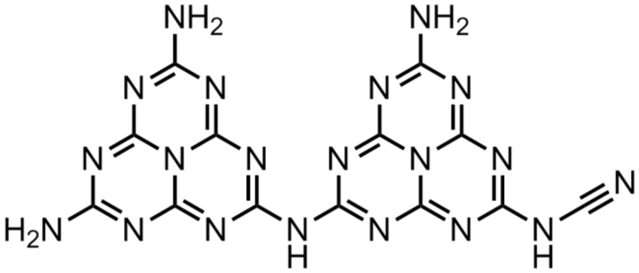

444

Supplementary Table 1: Possible assignment of some signals in the MALDI-TOF spectra from Supplementary Figure 3.

**Photocatalyst**	**Catalyst loading (mg mL^{-1})**	**AQE (%)**	**Wavelength (nm)**	**Reactant solution**
Pt/KSCN-treated melon (first 6 h)	1	10	400	MeOH
Pt/KSCN-treated melon (steady rate)	1	9	400	MeOH
Pt/Melon (this work)	1	0.5	400	MeOH
Pt/Melem oligomer[7]	1	1	400	MeOH
Pt/Poly(triazine imide)[8]	1	Inactive	-	MeOH
Pt/Poly(triazine imide)[8]	1	0.6	400	Triethanolamine
Pt/Poly(triazine imide), 4-amino-2,6-dihydroxypyrimidine doped[8]	1	0.3	400	MeOH
Pt/Poly(triazine imide), 4-amino-2,6-dihydroxypyrimidine doped[8]	1	3	400	Triethanolamine
Pt/Azine COF[9]	0.5	Inactive	-	MeOH
Pt/Azine COF[9]	0.5	0.5	450	Triethanolamine
Pt/Melon (urea)[10]	0.43	27	400	Triethanolamine
Pt/Melon hollow nanosphere[11]	0.2	7.5	420	Triethanolamine
Pt/Poly(triazine imide), 2,4,6-triaminopyrimidine doped[12]	1	15	400	Triethanolamine
Pt/CdS[13]	26	8.2	436	Na_2SO_3
Pt/$AgInZn_7S_9$[14]	1	20	420	$Na_2S + K_2SO_3$
$Rh_{2-x}Cr_xO_3$ / $(Ga_{0.88}Zn_{0.12})(N_{0.88}O_{0.12})$[15]	0.81	6	420-440	H_2SO_4 (pH 4.5)

Supplementary Table 2: Comparison of the photocatalytic activity for hydrogen evolution, based on AQE values, of the carbon nitrides synthesized in this work and selected visible light active photocatalysts from our group and from the literature.

Signal number	Chemical shift (ppm)	Relaxation Time T_1 (s)	Assignment	Area Ratio
C1	118.2	>1000	$N\underline{C}N^-$	**
C2	156.9	169 ±16	$\underline{C_3}N_c$	3.00*
C3	163.3	159 ±11	$\underline{C}$–NH–$\underline{C}$	1.67
C4	167.6	176 ±22	$\underline{C}$–NCN^- H^+	1.14
C5	171.8	163 ±21	$\underline{C}$–NCN^- K^+	0.198
N1	-275	>2500	–$\underline{N}$–C≡N	**
N2	-241	384 ±27	–NH–	1*
N3	-225	444 ±28	N_c	0.85
N4	-184	195 ±16	C=N	2.95
N5	-178	†	–N–C≡$\underline{N}$	**

*Integral used for normalization

**Not calculated, due to unreliability of the integral

†Not determined due to signal overlap

Supplementary Table 3: Assignment and quantification of the solid state MAS ^{13}C NMR spectra of the KSCN-treated melon. To check the validity of this quantification, the sum of the integration ratios of the peripheral carbons (C3-5) is 3.01 and is nearly identical to the expected value of 3.0, the area of the three inner carbons (C2) of the heptazine. Quantification of the NCN integral is unreliable as the relaxation time of this carbon appears to be in excess of 1000 s.

Element	KSCN-treated Melon		Amorphous Melon	
	wt%	mol%	wt%	Mol%
C	26.66 ±0.02	27.30	35.40 ±0.08	32.39
N	44.36 ±0.09	38.94	60.6 ±0.5	47.52
H	2.55 ±0.04	31.31	1.83 ±0.05	20.09
K	7.54 ±0.09	2.38	-	
S	0.17 ±0.10	0.07	-	
**Total**	**81.0 ±0.2**		**97.8 ±0.5**	
**Calculated Molecular Formula**		$C_7N_{10}K_{0.6}$		$C_{6.1}N_9$
**Postulated Structure**	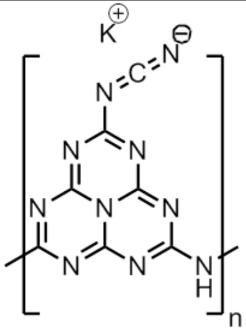 0.6	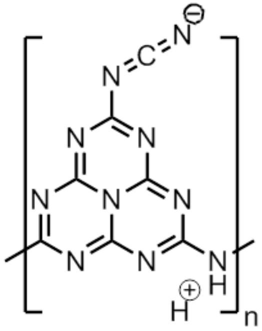 0.4	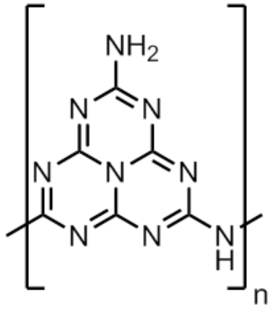	

Supplementary Table 4: Elemental analyses of KSCN-treated and untreated melon. All figures and uncertainties are averages and standard deviations of three replicates. Note that, for the KSCN-treated melon, there is around 23 wt% of water based on the TGA profile (Supplementary Figure 22). Hydrogen was not used in the calculation of molecular formulae due to the large uncertainty in determining water content.

	BET surface area (m^2 g^{-1})	Pore volume (cm^3 g^{-1})	Pore width (nm)	H_2 evolution rate† (µmol h^{-1})	H_2 evolution rate normalized to BET surface area (µmol h^{-1} m^{-2})	Apparent quantum efficiency‡ (%)
Melon	16	0.056*	3.2*	2.0	6.3	0.5
KSCN-treated melon	55	0.15**	1.9**	24.7	22.5	9.3

*Calculated using carbon-type sorbent with slit pores, NLDFT equilibrium model, fitting error 2.5%

**Calculated using carbon-type sorbent with cylindrical pores, QSDFT model fitted to adsorption branch, fitting error 0.49%

†Under AM1.5 irradiation; value here refers to the stable hydrogen evolution rate

‡Irradiated with full spectrum of xenon lamp with 400 nm band pass filter (40 nm FWHM) at intensity of 32 mW (10 mW cm^{-2})

Supplementary Table 5: Summary of morphological analyses and photocatalytic activities of the KSCN-treated melon, compared with amorphous melon.

Element	Pristine		After photocatalysis	
	wt%	mol%	wt%	mol%
C	26.66 ±0.02	27.30	27.55 ±0.09	27.71
N	44.36 ±0.09	38.94	43.63 ±0.08	37.62
H	2.55 ±0.04	31.31	2.87 ±0.04	34.33
K	7.54 ±0.09	2.38	1.11**	0.34
S	0.17 ±0.10	0.07	*	
Pt	*	*	5.33**	
**Total**	**81.0 ±0.2**		**80.5 ±0.1**	
**Calculated Molecular Formula**		$C_7N_{10}K_{0.6}$		$C_{7.4}N_{10}K_{0.09}$

*Not measured

**Insufficient sample for multiple analyses for calculating standard deviation

Supplementary Table 6: Elemental analysis before and after photocatalysis. Note that, since only 20 mg was used for each photocatalytic experiment, not every element could be analysed in replicates to determine the standard deviation.

	HOMO-1	HOMO	LUMO	LUMO+1	ΔE
**PBE**					
–NH_2	-5.797	-5.795	-3.1544	-2.967	2.641
–NCNH	-6.754	-6.743	-4.170	-4.034	2.573
–NCN^-K^+	-5.016	-4.896	-3.390	-3.023	1.506
**PBE0**					
–NH_2	-6.835	-6.834	-2.454	-2.245	4.380
–NCNH	-7.854	-7.802	-3.510	-3.359	4.292
–NCN^-K^+	-6.049	-5.923	-2.710	-2.334	3.213
**ic-PBEh**					
–NH_2	-8.852	-8.848	-1.075	-0.841	7.773
–NCNH	-9.873	-9.810	-2.163	-1.994	7.647
–NCN^-K^+	-8.053	-7.921	-1.361	-1.246	6.560

Supplementary Table 7: Energy levels for HOMO-1, HOMO, LUMO, and LUMO+1, as well as the HOMO-LUMO energy difference for the pentamers in Supplementary Figure **25** to Supplementary Figure **27** with the three different terminations, calculated using the different functionals (PBE, PBE0, and ic-PBEh). All values have units in eV and scaled to the vacuum level.

Supplementary Discussion

Analyses of crystalline and amorphous melem and melon

The key differentiator between crystalline and amorphous melem/melon is the presence of evolved volatile products during the condensation process, which are mostly ammonia and some hydrogen cyanide. For the crystalline product, carrying out the synthesis in ampoules traps these gases, which reversibly break down and reform the polymer, thus leading to crystalline products as defects are healed in the resulting formation/decomposition equilibrium. The decomposition, and hence the defect healing process, is absent in an open synthesis i.e. when the gaseous products are not trapped, leading instead to kinetically stable but amorphous products. This aspect is obvious when comparing the IR spectra of crystalline and amorphous melem (Supplementary Figure 2), where the latter contains an additional strong absorption at around 1215 cm^{-1}, attributed to further condensations at the primary amines of melem to secondary or tertiary amines. In the case of melon, TGA-MS analyses demonstrate that HCN and CO_2 are evolved at a lower temperature for the amorphous one than for the crystalline counterpart (Supplementary Figure 2). Evolution of HCN has been alluded to the presence of incompletely condensed termini present as cyanamide,[16] while CO_2 evolution at the decomposition temperature suggests oxygen-bearing group(s) are attached to the heptazine core. Evidence for the presence of incompletely cyclized heptazines can be found in the MALDI-TOF spectra of amorphous melem (Supplementary Figure 3; assignment in Supplementary Table 1: Possible assignment of some signals in the MALDI-TOF spectra from Supplementary Figure 3.), which show the possible presence of cyanamide-terminated heptazines. Note that crystalline melon did not appear to ablate, as heptazine species (monomer, dimer etc) were not observed in their MALDI-TOF spectra. These characterizations strongly suggest that these functional groups are, in fact, native to amorphous melons and may be responsible for their activity. For this characterization, two different matrices were employed and only signals appearing in both cases are assigned so as to avoid spurious assignment of impurities in the matrices.

Selection of model photocatalysts

The near absence of activity in the crystalline products (Supplementary Figure 4), contrasted with the presence of activity in the amorphous counterparts, thus led us to consider the functional groups resulting from incomplete cyclization of the heptazine core or incorporation of carbon or oxygen impurities as possibly the catalytically relevant "defects". Alternatively, it is also possible that the amorphous products lack condensed packing compared to the crystalline counterparts, thus exposing the catalytically relevant groups that would otherwise be bound up by hydrogen bonding. An example of such groups could be the primary or secondary amines, or the Lewis basic nitrogen of the heptazine core. Therefore, to identify the functional group(s) responsible for the hydrogen evolution, we screened for photocatalytic activity using heptazine compounds as molecular models with the following functional groups at the 2, 5 and 8 positions: primary, secondary and tertiary amines (**1**, **4**, **5**), imide (**3**), keto/alcohol (**7**), and oxy- (**8**) groups. Crystalline melem is employed for the model **1** and for the synthesis of **3** to avoid spurious results due to the presence of melem oligomers present in amorphous melem. Heptazine molecules appended with a phenyl blocking group without and with a coordinating group (**9** & **10** respectively) were also used as a control to elucidate the necessity of a coordinating group. The diffuse reflectance spectra of all these models are shown in Supplementary Figure 5.

Lastly, potassium melonate (**6a**) as well as its complexes with transition and lanthanide metals (**6b-n**) were also included in the screening, since the cyanamide functionality can occur in incompletely condensed melon, as suggested in the heptazine cyclisation mechanism proposed in the literature (Supplementary Figure 1) as well as in the TGA-MS and MALDI-TOF results.[16,17] Metal complexes of this ligand may also yield insight into how charge is relayed from the heptazine to the hydrogen, possibly through the redox active metal centre. It is envisaged that, upon excitation, the heptazine core reduces the coordinated metal. The reduced metal in turn reduces or transfers the electron to the platinum in solution for hydrogen evolution, while the coordinated metal reverts to its original valence state. Characterisation of the meloantes are shown in Supplementary Figure 6 (FTIR), Supplementary Figure 7 (diffuse reflectance spectra), and Supplementary Figure 8 (XRD).

Screening of model photocatalysts for hydrogen evolution activity

The standard protocol for photocatalytic screening was performed under full spectrum of the xenon lamp using the model catalyst (20 mg), aqueous methanol (10 vol%, 20 mL) as the electron donor and platinum from the in-situ photoreduction of H_2PtCl_6 (≈1 wt%) as co-catalyst. Methanol was selected, rather than the commonly used triethanolamine, as this electron donor oxidises cleanly to CO_2 via formic acid, and no complicating side product (e.g. carbon monoxide) was observed under the conditions used. In these experiments, the models were only screened for the presence or absence of photocatalytic activity without the rates being compared, on account of their different spectral profile and solubility/dispersibility in the medium used. For example, potassium melonate (**5a**), potassium cyamelurate (**6**) and tri(*p*-benzoic acid)heptazine (**9**) are soluble in water, whereas all others have limited solubility and differing hydrophilicity/dispersibility. Moreover, for these screening experiments, we caution that absence of activity in a model does not necessarily exclude a specific functional group to be catalytically insignificant, since properties intrinsic to the 1D polymer phase (e.g. conformational flexibility and intra-/intermolecular interactions, particularly hydrogen bonding and heptazine-heptazine stacking interactions) can vary the carrier dynamics[18-20] as well as the HOMO/LUMO distribution.[7] Moreover, the asymmetry around the heptazine core in melon (two bridging 2° amines and one 1° amine per heptazine) may induce a dipole, a property that is known to mitigate electron-hole recombination.[21] Recently published work[22] on the transport mechanism indicates that, in carbon nitrides, the excitons dissociate into polarons that hop onto neighboring polymer strands in the stacking direction, with hopping dynamics improving as the stacking distance decreases. In contrast, all model photocatalysts have C3 symmetry, lack the close proximity between the heptazine cores even in the condensed phase, and are more subjected to exciton quenching by the solvent.[23,24] The exciton transport to the reaction center (platinum) is therefore expected to be less favored for photocatalysis in the molecular models than for the carbon nitrides. For these reasons, the subsequent discussions focus on the photocatalytically relevant functional groups independent of the properties specific to polymeric structures.

Activity was observed for the heptazine functionalized with the ethylamine (**2**), the oxyanion (**6** and **7**) and the benzoic acid (**9**) groups, while amongst the metal melonates, none is active with the exception of platinum(II) melonate (**6k**), the hydrogen evolution plot of which is shown in Supplementary Figure 11 and further analyses are presented in the next section. The commonality with all these active models is that moieties known to coordinate with platinum are present, making this a required but insufficient criterion to exhibit hydrogen evolution. Presumably the coordination acts as a bridge for electron transfer by an inner sphere mechanism. The absence of activity for the

water soluble potassium melonate, in contrast to the active but insoluble platinum(II) melonate, would suggest an unfavorable interaction based on the hard/soft ligand principle. The melonate readily forms an insoluble coordination compound with the softer Pt(II) salt $(NH_3)_4Pt(NO_3)_2$ due to the strong interaction between the soft cyanamide ligand on the melonate and the soft Pt(II) metal. This favourable interaction leads to more efficient charge transfer and is hence photocatalytically active, as consistent with the many Pt(II) complexes that also exhibit activity for hydrogen evolution.[25] In contrast, the cyanamide ligand interacts weakly with the high valent (hence harder) Pt(IV) in H_2PtCl_6, and thus no insoluble complex is formed. Coupled with the dilution factor in the screening protocol where the amount of H_2PtCl_6 is 51 µM, the weak interaction between the melonate and the platinum compound hinders the electron transfer from the photo-excited unit to the hydrogen evolving center. We can nonetheless force the potassium melonate to exhibit photocatalytic hydrogen evolution by using a much larger amount of H_2PtCl_6 to enhance the interaction probability. Using 12 times more H_2PtCl_6 than the standard protocol and conducting the photocatalytic experiment over tens of hours, we were able to observe hydrogen evolution from potassium melonate (Supplementary Figure 11). Note that the same results were obtained when we employed $Pt(NO_3)_4$ in place of H_2PtCl_6.

Absence of activity in all but the platinum melonate indicates that the coordinated metal does not act as a charge relay, which may be attributed to the following possibilities: 1) unfavorable contact between the platinum and the metal melonate for transfer of the photo-generated charge, or 2) inability of the photo-electron to reduce protons to hydrogen, caused by the decrease in potential energy of the excited states for the transition metal melonate complex. The first explanation is consistent with our conclusion that a coordinating group is required to act as a conduit for electron transfer from the light-absorbing heptazine core to the hydrogen evolving platinum center. When the cyanamide is coordinated to the transition metals or the lanthanides, this ligating group is blocked from interacting with the platinum species, thus preventing electron transfer for hydrogen evolution. We conducted some preliminary tests as to whether this deactivation applies for the active models, focusing on the potassium cyamelurate, which is photocatalytically active in the standard screening protocol (with H_2PtCl_6). Insoluble cyamelurate salts of chromium(III), zinc(II) and lanthanum(III) – all of which are expected to have coordination at the oxyanion – exhibited no activity under the screening conditions, thus supporting our hypothesis on coordinative blocking. The second explanation is based on the observation that complexation can lead to substantial changes in redox potentials. In this case, if the redox potential is significantly shifted in the anodic direction, it is possible that hydrogen evolution is no longer thermodynamically favoured. As a literature example of a ligand containing the NCN moiety, the dianionic 1,4-dicyanamidobenzene as a free ligand has the -1/-2 redox couple at -0.215 V vs NHE, while after coordination to Ru(III) this couple is greatly shifted anodically to +0.830 V vs NHE.[26]

Detailed analysis of platinum(II) melonate

The active complex, platinum melonate, showed stable activity over 70 h, even without the addition of H_2PtCl_6. Analysis of the spent catalyst showed that, while a small amount of Pt particles were formed as observed in TEM (Supplementary Figure 12), the majority of the compound remained unchanged as ascertained by XPS characterization (Supplementary Figure 13). Specifically, no discernable change was observed in the N 1s peaks and Pt 4f signals in platinum melonate before and after the photocatalytic reaction, with the Pt $4f_{7/2}$ peaks remaining at 73.3 eV, which

corresponds to Pt(II). The unchanging hydrogen evolution rate, after 70 h reaction, is indicative that the photocatalytic activity is not due to continuous Pt(0) formation, but rather originates from the Pt(II) centers. To identify whether the activity originated from Pt^{II} or metallic Pt^{0}, we attempted the experimental protocols proposed by Widegren and Finke.[27] These tests were however not clear-cut for photocatalytic reactions, as carbon disulfide competed with photoelectrons for proton reduction,[28] while mercury (and/or its oxide) are light scatterer, preventing photon collection from the platinum complex. Despite their unsuitability, evolution of trace amount of hydrogen was observable even when mercury was employed. Furthermore, XPS analyses of the spent catalysts did not detect the presence of PtHg amalgam (Figure S12), suggesting that platinum(II) complex was indeed the active component, rather than platinum(0). Slight shifts are however observable after mercury addition, specifically the shift from 73.32 to 73.03 eV for the Pt $4f_{7/2}$ signal and 99.90 to 100.93 eV for the Hg $4f_{7/2}$ signal. As these signal positions are almost identical to those of a bridged Pt^{II}–Hg^{0} heterometallic complex,[29] it is likely a similar complex formed in the present case.

Supporting discussions of the NMR spectra

We attribute the weak, broad cyanamide signals in the ^{13}C and ^{15}N NMR of KSCN-treated melon, even after enrichment, to the following possibilities, or combination thereof: (1) signal broadening due to the conformational flexibility of the cyanamide group, which in turn leads to different local environments, or due to differing cations (see below regarding elemental analysis) and/or cation coordination modes; (2) unfavorable cross-polarization dynamics in the CP experiments, consisting in a short proton $T_{1\rho}$ and very long cross-relaxation time T_{IS}, considering the very long relaxation time for atoms in this moiety (>1000 s for C, >2500 s for N, see Table S3); and (3) low quantity or insufficient enrichment of the cyanamide moiety. The first explanation is supported by the signal broadening in the Raman spectrum and is consistent with the 3 possible coordination geometries in potassium melonate.[30] The third explanation supports the notion[31] that melonate formation may not be as straightforward as the proposed mechanism in the literature (Scheme S2). One should bear in mind that melon can also be prepared from cyanamide-containing compounds (e.g. cyanamide or dicyandiamide) or from thiocyanate salts, with the mercury or ammonium salts being the historical precursors.[32] Based on the signal intensities in the ^{15}N NMR direct excitation spectrum, we observed that using the isotope-enriched KSCN as salt melt did not lead to exclusive enrichment of the cyanamide group, but instead, the enriched ^{15}N and ^{13}C are distributed, perhaps unevenly, over the entire polymer. This apparent scrambling effect may be rationalized as due to a decomposition/formation equilibrium in melon in the KSCN melt, where the heptazine units are broken down into cyanamide, while the isotope-enriched cyanamide groups cyclize into heptazines.

Information regarding polymer length, in theory, can be obtained from 1D direct excitation NMR experiments, since the magnetization of all carbon nuclei (except the cyanamide carbon due to its long relaxation time) of the polymer are fully restored (Table S3).[33] Assuming a linear polymer, the polymer length can be roughly estimated using the ratio of the integrals from the signals belonging to the carbons connected to the 2° bridging amines (C3) and the 3 core carbons (C2) with the formula:

$$\frac{C3}{C2} = \frac{2(n-1)}{3n} \qquad (1)$$

where n is the number of monomer units in the polymer and N_c is the central nitrogen of the heptazine. With an integral ratio of 0.536, we estimate each polymer strand to have five monomer units. Note that we have thus far considered this sample to be a polymer based on the position of the N_c signal in the ^{15}N NMR spectrum in comparison with that of crystalline melon, even though 5 monomeric units is rather small. We must therefore emphasize that the minimum number of monomeric units required to exhibit polymeric properties is still unknown, as it is still uncertain what the polymer lengths of crystalline and amorphous melon are. Non-linear polymers, though not ruled out, were not detected from spectroscopic techniques. While we cannot completely exclude triazine-type moieties due to their NMR spectral similarity with their heptazine counterpart, their presence would be inconsistent with the observed intensities of the ^{13}C NMR signals and elemental analyses. Furthermore, species such as poly(triazine imide) is not observed in the XRD nor in the FTIR based on the NH band in the 3200–3310 cm^{-1} region.[34]

The less than expected amount of potassium indicates partial cation exchange with protons, likely during the purification step. This is supported by an ^1H-^{13}C correlation NMR experiment (Figure S20), the spectrum of which shows, in addition to the 2° amine protons at 6–7 ppm, an acidic proton environment at 10–11 ppm. These acidic protons have a long range coupling solely to the amine-connected carbon (C3), suggesting that they are loosely bound to the polymer and most likely located in-between the polymer strands.

Characterization of the spent photocatalyst

The spent catalyst after the 100+ h of irradiation was recovered by centrifugation, washed with water and dried, then characterized by FTIR, XRD, XPS and MAS ssNMR. The polymer backbone remained unaffected as evidenced by the signals related to the heptazine units and the bridging 2° amine in the FTIR (1308, 1216 and 806 cm^{-1}), NMR (^{13}C: 164 and 158 ppm), and XPS (401.0 eV). All signals in ^{15}N NMR are slightly shifted downfield compared to the pristine sample, but nonetheless assignable to the heptazine polymer. The most important changes in the spent catalyst are the loss of intensity of the cyanamide-related signals in the FTIR (2210–2120 cm^{-1}) and NMR (^{13}C: 171, 168 and 118 ppm; 15N: -276 ppm), weakened XRD reflections corresponding to the in-plane periodicity, shifts in the sp^2 C_{1s} and N_{1s} XPS signals assigned to the heptazine core, and reduction in signal intensity of the K_{2p} XPS signal. Note that loss of the 171 and 168 ppm ^{13}C NMR signals prevented quantification of hydrolysis by ^{13}C ssNMR. These spectra are in fact nearly identical to melon terminated with urea, formed by acid hydrolysis of the cyanamide moieties in the KSCN-treated melon. In contrast, no change was discernable in the FTIR and XRD for amorphous melon, which was used as a comparison. In the Pt_{4f} XPS region, Pt^0 and Pt^{II} are identified in both of the spent catalyst, KSCN-treated melon and melon, with the Pt^{II} species assigned to the charge-compensating interface between the anionic polymer and the metallic Pt^0 as the platinum particles nucleate and grow on the material during the photo-reduction. In the XRD pattern, the stacking reflection of the spent catalyst is split in two, with the reflection in the lower angle being identical to the material formed by acid hydrolysis of cyanamide moieties. For the reflection at the higher angle, this is about 0.1° 2θ higher than the corresponding reflection in the pristine material, corresponding to a decrease in the stacking separation. We attribute this “densification” to exchange of the potassium for protons during the photocatalytic reaction resulting in possibly more favorable stacking interactions; loss of potassium is evidenced by elemental analyses () and decrease in the K2p XPS signals in the spent catalyst. Correlation of stacking separation with the radii of the incorporated ions are also observed

for the related carbon nitride compound, poly(triazine imide). In this material, when the incorporated anion changes from bromide to chloride to fluoride, this separation decreases from 3.52 to 3.38 to 3.32 Å.[35] Similarly, when the cation changes from lithium to proton, the separation decreases from 3.38 to 3.22 Å.[34,36]

Supplementary Methods

Solid state magic angle spinning NMR

Solid state ^{1}H, ^{13}C and ^{15}N magic angle spinning (MAS) NMR experiments were performed on a Bruker Avance-III 400 MHz instrument at the frequencies of 400, 100.61 and 40.53 MHz, respectively (B_0 = 9.4 T). Chemical shifts for ^{1}H and ^{13}C are referenced to tetramethylsilane (TMS, $\delta(^{1}H,^{13}C) = 0.0$), while ^{15}N is referenced to nitromethane ($\delta = 0$). Magic Angle Spinning (MAS) with spinning rates ranging between 10 and 12.5 kHz was used in all experiments on solids. ^{13}C spectra with cross-polarization (CP) were recorded with a ramped polarization mixing and SPINAL-64 proton decoupling (^{1}H RF field of 80 kHz).[37] Quantitative ^{13}C spectra were acquired in a direct excitation mode with relaxation delay between the consecutive scans set to 1200 s. This relaxation delay was at least 4 times the longest T_1 in all but the –N=C=N carbon sites in the materials studied, as estimated from preliminary relaxation time measurements. Between 160 and 256 scans were commonly accumulated, with all experiments accompanied by SPINAL-64 decoupling. ^{1}H-^{13}C frequency switched Lee-Goldburg hetero-nuclear correlation (FSLG HETCOR) experiments[38] were carried out with ramped cross polarization, ^{1}H RF =100 kHz, and spinning speed of 12.5 kHz. Short mixing times of 50 -150 μs were used to in order to avoid long range polarization transfer. A total of 128 increments were made in an indirect dimension (1H), with 560 acquisitions per increment. Deconvolution and integration of the solid state spectra were carried out using the Dmfit software version 20111221.[33]

Photocatalytic hydrogen evolution

Photocatalytic experiments were performed in a double-walled glass reactor, where the outer compartment is circulated with thermostated water (25 °C), as previously described.[7] The reactor was top-irradiated through a quartz window with a xenon lamp (Newport, 300 W) equipped with a water filter and a full spectrum mirror (2000 nm > λ > 200 nm). An air mass (AM) 1.5 filter was also used where specified. The spectra are shown in Supplementary Figure 9, together with the bandpass filters used for quantum efficiency estimates. For each experiment, the catalyst powder (20 mg) was suspended in a solution of water (18 mL), methanol (2 mL) and dihydrogen hexachloroplatinate (5 µL, 8 wt% aqueous solution, Aldrich), which forms the platinum cocatalyst from its *in*-situ reduction. This platinum amount yields a loading of around 1 wt%. Methanol oxidizes photochemically to carbon dioxide *via* the aldehyde and possibly formic acid:

$$2h^{+} + CH_3OH \rightarrow CH_2O + 2H^{+} \quad (2)$$

$$2h^{+} + CH_2O + H_2O \rightarrow HCO_2H + 2H^{+} \quad (3)$$

$$2h^{+} + HCO_2H \rightarrow CO_2 + 2H^{+} \quad (4)$$

$$\text{Overall: } CH_3OH + H_2O \rightarrow CO_2 + 3H_2 \quad (5)$$

The headspace was subjected to several cycles of evacuation and argon backfill prior to the experiment. In the course of the experiment, the headspace of the reactor was periodically sampled manually and the components were quantified by gas chromatography (Thermo Scientific TRACE GC Ultra) equipped with a TCD detector using argon as the carrier gas. Note that, upon irradiation of the photocatalyst suspension in aqueous methanol solution, the KSCN-treated melon changes colour from yellow to cyan or turquoise indicative of a long-lived excited state, which we will discuss in greater depths in a following publication. After the photocatalytic experiment, the catalyst was recovered by centrifugation, washed with water, then dried at 60 °C in vacuum. For quick optimization of platinum loading, the photocatalytic experiments were performed in disposable septum-capped glass vials containing the catalyst (10 mg), water (9 mL) and methanol (1 mL) and a variable amount of platinum. The vial was stirred whilst irradiated using a xenon lamp as above for 3 h, then the hydrogen in the headspace was quantified. Experiments for the estimation of quantum efficiency were conducted using band pass filters with band centers at 400 nm and 500 nm with full width half maximum of 50 nm (Thorlabs). Irradiance of the incident light in watts was measured using a thermopile (Thorlabs) and converted to photon flux using the integral of the transmission of the band pass filters. Apparent quantum efficiency (AQE) was then calculated as:

$$AQE\ [\%] = \frac{2 \times Hydrogen\ Evolution\ Rate\ [mol\ h^{-1}]}{Photon\ Flux\ [mol\ h^{-1}]} \qquad (6)$$

To differentiate whether the photocatalytic activity in platinum melonate originates from the platinum complex or from metallic platinum, we applied Finke's test as follows.[27] The sample was employed for photocatalytic hydrogen evolution as described above but without addition of H_2PtCl_6 to verify hydrogen evolution, before recovering the spent catalyst by removing the water and methanol on an evaporating dish under ambient conditions. The recovered catalyst (17 mg) was then returned to the reactor, suspended in water (18 mL) and elemental mercury (956 mg), then stirred overnight to allow amalgamation of the mercury with metallic platinum, if present. Methanol (2 mL) was added as the electron donor and the photocatalytic reaction was performed as before.

DFT calculations

Density-functional theory (DFT) calculations were performed with the FHI-aims all-electron electronic structure code.[39,40] Results in this work are based on accurate numerically tabulated atom-centered basis sets at the "tier 2" level of theory as described in Blum *et al.*[39] The remaining numerical settings are chosen according to FHI-aims' "tight" defaults, including overlapping atom-centered integration grids[41] with up to 434 grid points per spherical integration grid shell distributed on between 49 (H) and 73 (O) radial grid shells per atom, a maximum angular momentum l=6 for the expansion of the electrostatic potential, and radial extents of the basis functions up to 6 Å away from each nucleus. The exact-exchange operator in hybrid functionals is treated by a high-accuracy resolution of identity based implementation as described and benchmarked by Ren *et al.*[40] All molecular geometries used in this work were obtained by total-energy minimizations to the nearest local minimum of the potential-energy surface at the DFT-PBE[42]+vdW level of theory including the Tkatchenko-Scheffler[43] pairwise van der Waals correction. For all hybrid functional calculations of generalized Kohn-Sham orbitals and eigenvalues, the atomic positions were kept fixed at the DFT-PBE+vdW geometries. Orbitals and generalized Kohn-Sham single particle energies are calculated using an "internally consistent" approach[44] based on the PBE-hybrid[45] functional (called ic-PBEh), where the exact-exchange fraction α is adjusted such that the generalized Kohn-Sham ionization

potential matches the result from a G_0W_0 many-body perturbation calculation based on the PBEh functional with the same α. In the present work, we use α=0.6882 as found in reference [7] for the isolated melem molecule.

For the heptazine monomer models, calculated frontier orbitals and their energy levels based on the ic-PBEh functional are shown in Supplementary Figure 15.

HOMO/LUMO locations for heptazine pentamer models with different terminations but with the same pentamer and HOMO and LUMO orbitals were also obtained using the ic-PBEh functional, shown in Supplementary Figures 25-27 and in Supplementary Table 7. For comparison, Figures 25-27 and in Supplementary Table 7 also include generalized Kohn-Sham eigenvalues and orbitals calculated at the semilocal DFT-PBE[42] level of theory and at the commonly used PBE0[46] hybrid functional, defined by α=0.25. These comparative results show that the important qualitative electronic structure indicators derived in this paper – orbital shapes and relative ordering of the levels between different models – do not depend strongly on the details of the chosen density functional, strengthening our conclusions.

Supplementary References

1 Schwarzer, A., Saplinova, T. & Kroke, E. Tri-s-triazines (s-heptazines)—From a "mystery molecule" to industrially relevant carbon nitride materials. *Coord. Chem. Rev.* **257**, 2032-2062 (2013).

2 May, H. Pyrolysis of Melamine. *J. Appl. Chem.* **9**, 340-344 (1959).

3 Hosmane, R. S., Rossman, M. A. & Leonard, N. J. Synthesis and structure of tri-s-triazine. *J. Am. Chem. Soc.* **104**, 5497-5499 (1982).

4 Shahbaz, M. *et al.* Tri-s-triazine: synthesis, chemical behavior, and spectroscopic and theoretical probes of valence orbital structure. *J. Am. Chem. Soc.* **106**, 2805-2811 (1984).

5 Makowski, S. J., Schwarze, A., Schmidt, P. J. & Schnick, W. Rare-Earth Melonates $LnC_6N_7(NCN)_3{\cdot}xH_2O$ (Ln = La, Ce, Pr, Nd, Sm, Eu, Tb; x = 8–12): Synthesis, Crystal Structures, Thermal Behavior, and Photoluminescence Properties of Heptazine Salts with Trivalent Cations. *Eur. J. Inorg. Chem.*, 1832-1839 (2012).

6 Sattler, A. & Schnick, W. On the Formation and Decomposition of the Melonate Ion in Cyanate and Thiocyanate Melts and the Crystal Structure of Potassium Melonate, $K_3[C_6N_7(NCN)_3]$. *Eur. J. Inorg. Chem.*, 4972-4981 (2009).

7 Lau, V. W.-h. *et al.* Low molecular-weight carbon nitrides for solar hydrogen evolution. *J. Am. Chem. Soc.* **137**, 1064-1072 (2015).

8 Schwinghammer, K. *et al.* Triazine-based Carbon Nitrides for Visible-Light-Driven Hydrogen Evolution. *Angew. Chem. Int. Ed.* **52**, 2435-2439 (2013).

9 Vyas, V. S. *et al.* A tunable azine covalent organic framework platform for visible light-induced hydrogen generation. *Nat. Commun.* **6**, 8508 (2015).

10 Martin, D. J. *et al.* Highly Efficient Photocatalytic H2 Evolution from Water using Visible Light and Structure-Controlled Graphitic Carbon Nitride. *Angew. Chem.* **53**, 9240-9245 (2014).

11 Sun, J. *et al.* Bioinspired hollow semiconductor nanospheres as photosynthetic nanoparticles. *Nat. Commun.* **3**, 1139 (2012).

12 Bhunia, M. K., Yamauchi, K. & Takanabe, K. Harvesting Solar Light with Crystalline Carbon Nitrides for Efficient Photocatalytic Hydrogen Evolution. *Angew. Chem. Int. Ed.* **53**, 11001-11005 (2014).

13 Matsumura, M., Saho, Y. & Tsubomura, H. Photocatalytic Hydrogen Production from Solutions of Sulfite Using Platinized Cadmium Sulfide Powder *J. Phys. Chem.* **87**, 3807-3808 (1983).

14 Tsuji, I., Kato, H., Kobayashi, H. & Kudo, A. Photocatalytic H_2 Evolution Reaction from Aqueous Solutions over Band Structure-Controlled $(AgIn)_xZn_{2(1-x)}S_2$ Solid Solution Photocatalysts with Visible-Light Response and Their Surface Nanostructures. *J. Am. Chem. Soc.* **126**, 13406-13413 (2004).

15 Maeda, K., Teramura, K. & Domen, K. Effect of post-calcination on photocatalytic activity of $(Ga_{1-x}Zn_x)(N_{1-x}O_x)$ solid solution for overall water splitting under visible light. *J. Catal.* **254**, 198-204 (2008).

16 Bojdys, Michael J. *On new allotropes and nanostructures of carbon nitrides* PhD thesis, Max-Planck Institut für Kolloid- und Grenzflächenforschung, (2009).

17 Algara-Siller, G. *et al.* Triazine-Based, Graphitic Carbon Nitride: a Two-Dimensional Semiconductor. *Angew. Chem. Int. Ed.* **53**, 7450–7455 (2014).

18 Tyborski, T. *et al.* Tunable optical transition in polymeric carbon nitrides synthesized via bulk thermal condensation. *J. Phys. Condens. Matter* **24**, 162201 (2012).

19 Merschjann, C. *et al.* Photophysics of polymeric carbon nitride: An optical quasimonomer. *Phys. Rev. B* **87**, 205204 (2013).

20 Tyborski, T. *et al.* Crystal structure of polymeric carbon nitride and the determination of its process-temperature-induced modifications. *J. Phys. Condens. Matter* **25**, 395402 (2013).

21 Li, L., Salvador, P. A. & Rohrer, G. S. Photocatalysts with internal electric fields. *Nanoscale* **6**, 24-42 (2014).

22 Merschjann, C. *et al.* Complementing Graphenes: 1D Interplanar Charge Transport in Polymeric Graphitic Carbon Nitrides. *Adv. Mater.* **27**, 7993-7999 (2015).

23 Magde, D., Rojas, G. E. & Seybold, P. G. Solvent Dependence of the Fluorescence Lifetimes of Xanthene Dyes. *Photochem. Photobiol.* **70**, 737-744 (1999).

24 Frank, H. A. *et al.* Effect of the Solvent Environment on the Spectroscopic Properties and Dynamics of the Lowest Excited States of Carotenoids. *J. Phys. Chem. B* **104**, 4569-4577 (2000).

25 Ozawaa, H. & Sakai, K. Photo-hydrogen-evolving molecular devices driving visible-light-induced water reduction into molecular hydrogen: structure–activity relationship and reaction mechanism. *Chem. Commun.* **47**, 2227-2242 (2011).

26 Rezvani, A. R., Evans, C. E. B. & Crutchley, R. J. Strong Metal-Metal Coupling in a Dinuclear (Terpyridine)(bipyridine)ruthenium Mixed-Valence Complex Incorporating the Bridging Ligand 1,4=Dicyanamidobenzene Dianion. *Inorg. Chem.* **34**, 4600-4604 (1995).

27 Widegren, J. A. & Finke, R. G. A review of the problem of distinguishing true homogeneous catalysis from soluble or other metal-particle heterogeneous catalysis under reducing conditions. *J. Mol. Catal. A: Chem.* **198**, 317-341 (2003).

28 Hurley, M. F. & Chambers, J. Q. Electrochemical reduction of carbon disulfide. Synthesis of carbon sulfide heterocycles. *J. Org. Chem.* **46**, 775-778 (1981).

29 Aw, B. H., Looh, K. K., Chan, H. S. O., Tan, K. L. & Hor, T. S. A. X-Ray photoelectron spectroscopic characterization of $[\{Pt(PPh_3)_2(\mu_3\text{-}S)\}_2PtCl_2]$, $[\{Pt_2(PPh_3)_4(\mu_3\text{-}S)_2Cu\}_2(\mu\text{-}dppf)][PF_6]_2[dppf = Fe\ (C_5H_4PPh_2)_2]$ and other heterometallic aggregates derived from $[\{Pt(PPh_3)_2(\mu\text{-}S)\}_2]$. *J Chem. Soc. Dalton Trans.*, 3177-3182 (1994).

30 Horvath-Bordon, E., Kroke, E., Svoboda, I., Fuess, H. & Riedel, R. Potassium melonate, $K_3[C_6N_7(NCN)_3].5H_2O$, and its potential use for the synthesis of graphite-like C3N4 materials. *New J. Chem.* **29**, 693-699 (2005).

31 Sattler, A. *Investigations into s-Heptazine-Based Carbon Nitride Precursors* Ludwig Maximilians University, (2010).

32 Liebig, J. Ueber die Zersetzung des Schwefelcyanammoniums und die Constitution der Schwefelblausäure. *Ann. Chem.* **53**, 330-348 (1845).

33 Massiot, D. *et al.* Modelling one- and two-dimensional solid-state NMR spectra. *Magn. Reson. Chem.* **40**, 70-76 (2002).

34 Wirnhier, E. *et al.* Poly(triazine imide) with Intercalation of Lithium and Chloride Ions [$(C_3N_3)_2(NH_xLi_{1-x})_3$·LiCl]: A Crystalline 2D Carbon Nitride Network. *Chem. Eur. J.* **17**, 3213-3221 (2011).

35 Chong, S. Y. *et al.* Tuning of gallery heights in a crystalline 2D carbon nitride network. *J. Mater. Chem. A* **1**, 1102-1107 (2013).

36 Zhang, Z. *et al.* High-Pressure Bulk Synthesis of Crystalline $C_6N_9H_3$·HCl: A Novel C_3N_4 Graphitic Derivative. *J. Am. Chem. Soc.* **123**, 7788-7796 (2001).

37 Fung, B. M., Khitrin, A. K. & Ermolaev, K. An Improved Broadband Decoupling Sequence for Liquid Crystals and Solids. *J. Magn. Reson.* **142**, 97-101 (2000).

38 Rossum, B.-J. v., Förster, H. & Groot, H. J. M. d. High-Field and High-Speed CP-MAS^{13}C NMR Heteronuclear Dipolar-Correlation Spectroscopy of Solids with Frequency-Switched Lee–Goldburg Homonuclear Decoupling. *J. Magn. Reson.* **124**, 516-519 (1997).

39 Blum, V. *et al. Ab initio* molecular simulations with numeric atom-centered orbitals. *Comput. Phys. Commun.* **180**, 2175-2196 (2009).

40 Ren, X. *et al.* Resolution-of-identity approach to Hartree–Fock, hybrid density functionals, RPA, MP2 and GW with numeric atom-centered orbital basis functions. *New J. Phys.* **14**, 053020 (2012).

41 Havu, V., Blum, V., Havu, P. & Scheffler, M. Efficient O(N) integration for all-electron electronic structure calculation using numeric basis functions. *J. Comput. Phys.* **228**, 8367-8379 (2009).

42 Perdew, J. P., Burke, K. & Ernzerhof, M. Generalized Gradient Approximation Made Simple. *Phys. Rev. Lett.* **77**, 3865-3868 (1996).

43 Tkatchenko, A. & Scheffler, M. Accurate Molecular Van Der Waals Interactions from Ground-State Electron Density and Free-Atom Reference Data. *Phys. Rev. Lett.* **102**, 073005 (2009).

44 Atalla, V., Yoon, M., Caruso, F., Rinke, P. & Scheffler, M. Hybrid density functional theory meets quasiparticle calculations: A consistent electronic structure approach. *Phys. Rev. B* **88**, 165122 (2013).

45 Perdew, J. P., Ernzerhof, M. & Burke, K. Rationale for mixing exact exchange with density functional approximations. *J. Chem. Phys.* **105**, 9982-9985 (1996).

46 Adamo, C. & Barone, V. Toward reliable density functional methods without adjustable parameters: The PBE0 model. *J. Chem. Phys.* **110**, 6158-6170 (1999).
